# Hypoxia and hypoxia-inducible factor (HIF) downregulate antigen-presenting MHC class I molecules limiting tumor cell recognition by T cells

**DOI:** 10.1371/journal.pone.0187314

**Published:** 2017-11-20

**Authors:** Shalini Sethumadhavan, Murillo Silva, Phaethon Philbrook, Thao Nguyen, Stephen M. Hatfield, Akio Ohta, Michail V. Sitkovsky

**Affiliations:** 1 New England Inflammation and Tissue Protection Institute, Northeastern University, Boston, MA, United States of America; 2 Dana Farber Cancer Institute, Harvard Institutes of Medicine, Boston, MA, United States of America; University of Thessaly Faculty of Medicine, GREECE

## Abstract

Human cancers are known to downregulate Major Histocompatibility Complex (MHC) class I expression thereby escaping recognition and rejection by anti-tumor T cells. Here we report that oxygen tension in the tumor microenvironment (TME) serves as an extrinsic cue that regulates antigen presentation by MHC class I molecules. In support of this view, hypoxia is shown to negatively regulate MHC expression in a HIF-dependent manner as evidenced by (i) lower MHC expression in the hypoxic TME *in vivo* and in hypoxic 3-dimensional (3D) but not 2-dimensional (2D) tumor cell cultures *in vitro*; (ii) decreased MHC in human renal cell carcinomas with constitutive expression of HIF due to genetic loss of von Hippel-Lindau (VHL) function as compared with isogenically paired cells with restored VHL function, and iii) increased MHC in tumor cells with siRNA-mediated knockdown of HIF. In addition, hypoxia downregulated antigen presenting proteins like TAP 1/2 and LMP7 that are known to have a dominant role in surface display of peptide-MHC complexes. Corroborating oxygen-dependent regulation of MHC antigen presentation, hyperoxia (60% oxygen) transcriptionally upregulated MHC expression and increased levels of TAP2, LMP2 and 7. In conclusion, this study reveals a novel mechanism by which intra-tumoral hypoxia and HIF can potentiate immune escape. It also suggests the use of hyperoxia to improve tumor cell-based cancer vaccines and for mining novel immune epitopes. Furthermore, this study highlights the advantage of 3D cell cultures in reproducing hypoxia-dependent changes observed in the TME.

## Introduction

The tumor microenvironment (TME) has been identified as a critical factor in controlling the progression and metastasis of cancer, along with dictating responses to conventional anti-tumor therapies [1–3]. In particular, hypoxia and the hypoxia-inducible factor-1α (HIF-1α) can be tumor-protective in many solid tumors and thus be a poor prognosis factor [4]. Despite significant advances in understanding of how hypoxia leads to an immunosuppressive TME by inhibiting effector functions of immune cells [5–7], little is known about the mechanisms by which hypoxia alters tumor immunogenicity [8–10].

Immunogenicity is critical in the establishment of an effective adaptive immune response [11, 12] and is largely dependent on tumor-associated antigen presentation by Major Histocompatibility Complex (MHC) class I molecules [13]. Altered or complete loss of MHC class I expression has been found on 60–90% of human cancers of different histological origins such as head and neck, skin, breast and lung [14, 15]. Cancers with downregulated MHC expression have been associated with greater metastatic potential and present a poor prognosis for patients [16, 17]. Clinically, the nature of the preexisting MHC defects in cancers has a crucial impact on determining the final outcome of cancer immunotherapy [18, 19]. Thus, there is an acute medical need to improve tumor immunogenicity [20, 21] and prevent evasion of tumors from tumor-reactive T cells by increasing our understanding of the complex regulation of MHC class I expression and developing methods to manipulate it [22, 23].

MHC class I expression is primarily regulated transcriptionally by integration of tissue-specific intrinsic signals as well as extrinsic signals from the microenvironment [24, 25]. In addition, the density of the peptide-MHC complexes at the surface can be affected by the efficiency of the antigen processing machinery (APM) that generate and transport antigenic peptides [26–28]. The heterodimeric complex transporter associated proteins (TAP) that shuttles peptides from cytosol into the ER and components of the immunoproteosome low molecular weight protein 2 and 7 (LMP2 and LMP7) that generate peptides from proteins have been shown to be potential targets of von Hippel-Lindau tumor suppressor (pVHL)[9], a protein that directs substrates (including HIF) to proteasomal degradation under normoxic conditions [29, 30].

In this study, we focused on the regulation of MHC class I expression on tumors and found a positive correlation between local tissue oxygen tension and levels of MHC. Mechanistic studies showed a HIF-dependent MHC downregulation in hypoxic environments. In addition, oxygen may affect the density of peptide-MHC complexes at the cell surface by regulating TAPs and LMPs.

## Results

### Hypoxia mediates down-regulation of MHC class I expression *in vivo*

**Fig 1:**
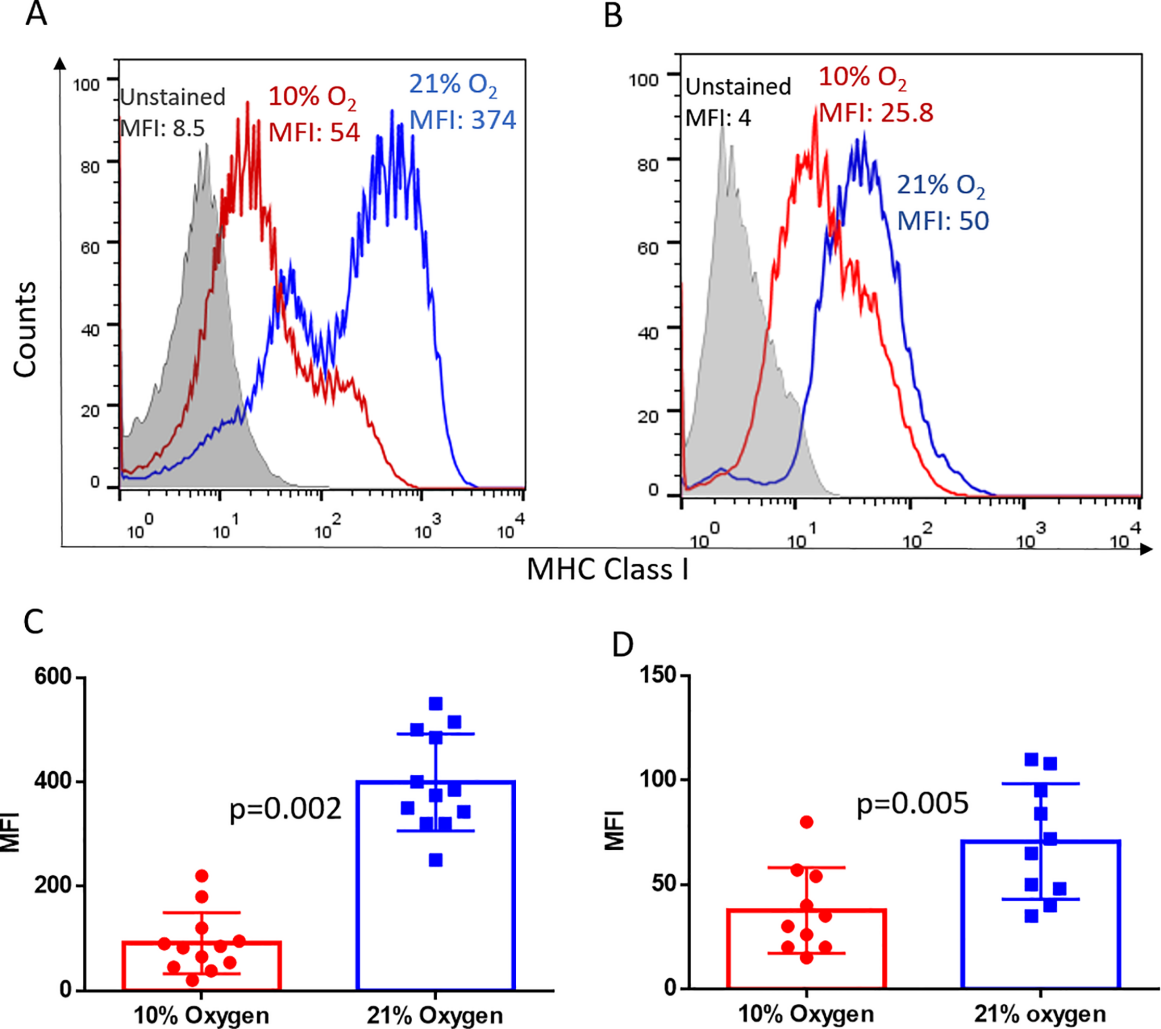
Hypoxia downregulates MHC class I expression on tumor cells *in vivo*. Whole body exposure of mice with 11 day established MCA205 pulmonary tumors (**A**, **C**; n = 6 per group per experiment) or 15 day established subcutaneous tumors (**B**, **D**; n = 5 per group per experiment) to 10% oxygen for 48h significantly downregulated MHC class I expression on tumor cells as compared with mice breathing 21% oxygen. MHC class I levels were determined by flow cytometry. Representative histograms (**A and B**) and associated quantification and statistics (**C and D**) of 2 independent experiments are shown. The significance of differences was analyzed by the Student’s t-test (two-sided); p = 0.002 (C), p = 0.005 (D). Grey filled: Unstained control; Red: Hypoxia; Blue: Normoxia. MFI: mean fluorescence Intensity. Error bars indicate SD.

Our previous findings show that MHC class I is heterogeneously expressed in the TME, with hypoxic regions having lower MHC expression as compared with normoxic regions of the same tumor [31]. Building upon these observations, we hypothesized that oxygen tension in the tissue microenvironment may regulate the extent of MHC class I expression in tumors. To test this, mice with established MCA-205 sarcoma were exposed to either respiratory hypoxia (10% O_2_) or normoxia (21% O_2_) for 48h, followed by examination of changes in MHC expression. We show that, independent of anatomical location, both pulmonary tumors (Fig 1A and 1C) and subcutaneous solid tumors (Fig 1B and 1D) from hypoxia breathing mice had significantly downregulated expression of MHC class I molecules as compared with tumors from mice in normoxic conditions.

### Three-dimensional (3D) cell culture systems recreate the biological effects of intra-tumoral hypoxia

**Fig 2:**
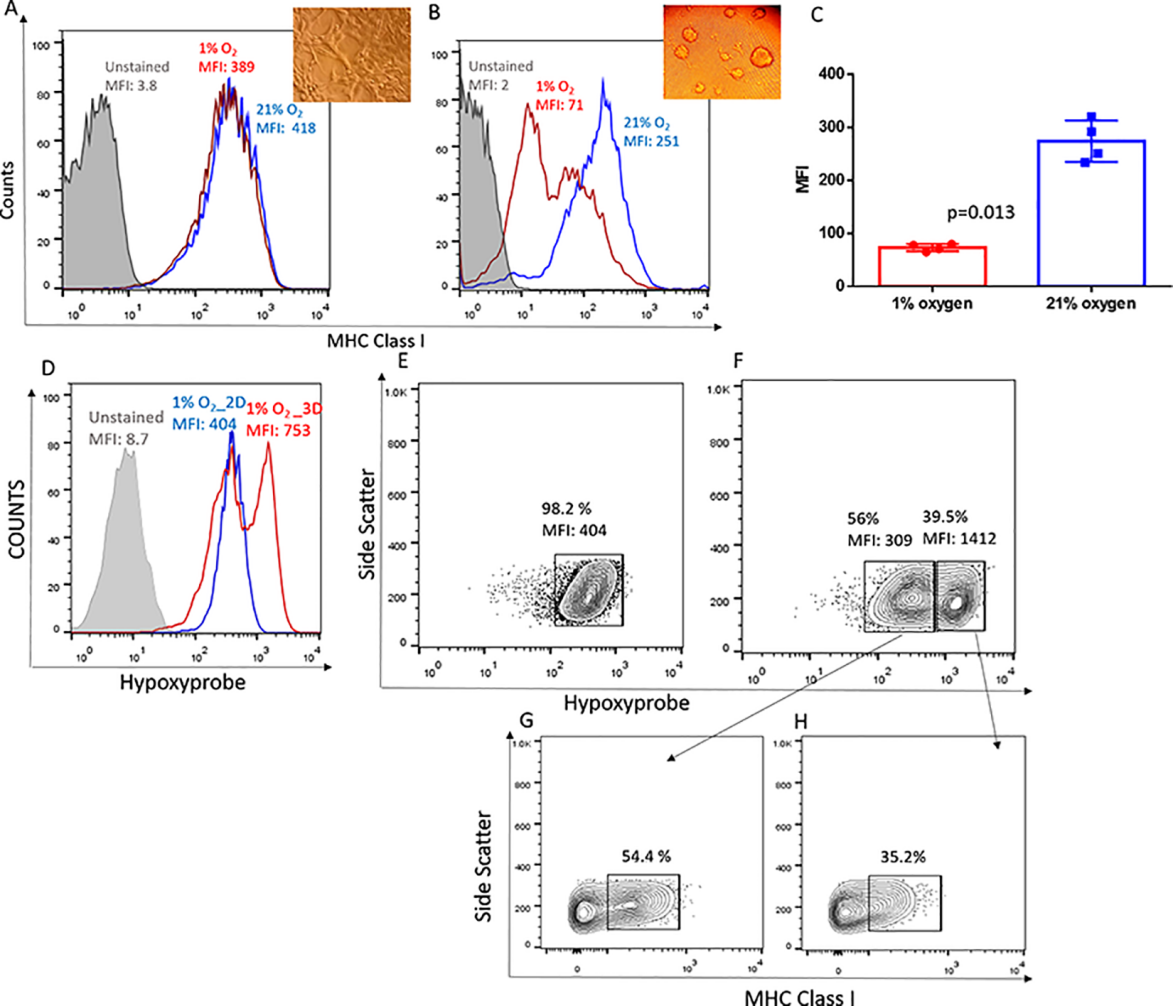
Hypoxia downregulates MHC class I expression *in vitro* in 3D but not in 2D culture systems and requires the deeper hypoxia achieved in the 3D system. **(A-C):** MCA205 tumor cells were cultured as 2D monolayers **(A)** or as 3D spheroids **(B,C)** and cultured under 21% O_2_ or 1% O_2_ for 48h. Levels of MHC class I expression was determined using flow cytometry. Representative histograms **(B)** and associated quantification and statistics **(C)** of 4 independent experiments are shown. The significance of differences was analyzed by the Student’s t-test (two-sided); p = 0.013 (C). Grey filled: Unstained control; Red: Hypoxia; Blue: Normoxia. MFI: mean fluorescence intensity. Inset: 40X magnification of MCA205 grown at 1% O_2_ in 2D culture **(A)** or in 3D culture **(B)**. Error bars indicate SD. **(D)** Representative flow cytometry histograms of Hypoxyprobe-1 (HP) indicating significantly increased levels of hypoxia in MCA205 cells grown under 1% oxygen for 48h as 3D spheroids (Red histogram) as compared with 2D monolayers (Blue histogram). n = 4. **(E)** Contour plots representing intensity of hypoxia within MCA205 cultures grown as 2D monolayers show 98% of the population was intermediately hypoxic. **(F)** MCA205 cells grown as 3D spheroids show two distinct populations of intermediately hypoxic (56%; HP MFI = 309) and severely hypoxic (39.5%; HP MFI: 1412) regions. **(G, H)** Gating on the 2 distinct hypoxic populations in the spheroid revealed inverse correlation between MHC class I and hypoxia levels. Less hypoxic cells had significantly higher percentage of MHC class I positive cells **(G)** and more hypoxic regions had lower percentage of MHC class I positive cells **(H)**. **(D-H)** Representative data of 4 independent experiments.

Next, we intended to delineate the molecular mechanisms by which hypoxia downregulated MHC class I expression using *in vitro* cell culture systems. However, standard monolayer tumor cultures unexpectedly failed to recapitulate the effects of hypoxia on MHC class I molecules observed *in vivo* (Fig 2A). This interpretation prompted us to re-assess the current cell culture system and led us to systematically test different media compositions and culture formats (data not shown). We found that the 3D culture systems accurately replicated the effects of hypoxia on the downregulation MHC class I (H-2Kb, H-2Kd) expression, similar to what we observed *in vivo* (Fig 2B and 2C and S1A–S1F Fig). Thus, while routine 2D cultures have been very valuable to address many experimental questions in immunology, they do not mimic the physiological hypoxic microenvironments found in *in vivo* tumors [32]. Hypoxia was found to downregulate MHC expression in both 2D and 3D cultures of murine thymomas EL4, since this cell line tends to grow as small 3D clumps *in vitro* even in typical 2D culture plates (S1G and S1H Fig).

### The lower oxygen tension achieved in 3D culture systems is necessary for hypoxia-mediated MHC class I downregulation

To explain the differential expression of MHC class I in 2D and 3D cultures, we hypothesized that variations in levels of hypoxia may occur within each culture system. To test this, we used the well-documented marker of hypoxia, Hypoxyprobe-1 [33]. We found that MCA205 cells grown as 3D spheroids under 1% O_2_ were significantly more hypoxic, as seen by increased Hypoxyprobe staining, compared to their 2D counterparts cultured under the same conditions (Fig 2C). Based on the Hypoxyprobe staining, two distinct populations of tumor cells–Hypoxyprobe high (39.5%) and Hypoxyprobe low (56%), could be identified in the spheroids whereas the entire population of cells was equally hypoxic when grown as a monolayer in 2D culture plates (Fig 2C–2E). Similarly, no hypoxic regions could be identified in 2D cultures grown under normoxic conditions (21% O_2_), whereas 16% of the population of cells from spheroid cultures was hypoxic, even under normoxic conditions (S2A, S2B and S2D Fig).

An inverse correlation between MHC class I expression and the levels of hypoxia within the cells was observed in the 3D spheroids (i.e., Hypoxyprobe^high^ = MHC^low^), further confirming our observations of hypoxia-based downregulation of antigen presentation (Fig 2E–2G, S2C and S2E Fig).

### Down-regulation of MHC class I by hypoxia is associated with less recognition and killing of tumor cells by cytotoxic T lymphocytes

**Fig 3:**
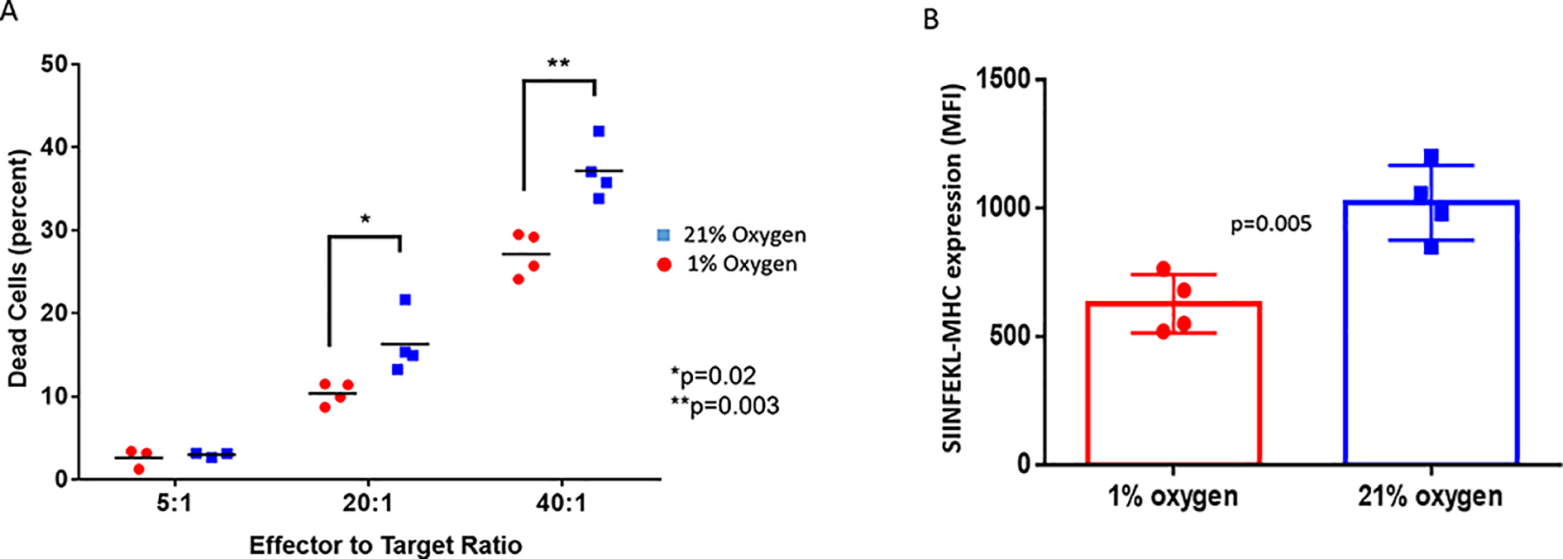
Hypoxia-mediated downregulation of MHC class I expression impairs recognition and killing of tumor cells by CTLs. (**A**) Hypoxic-grown MCA205-OVA cells with downregulated MHC class I expression were poorly recognized and killed by effector OT.1 T cells compared to normoxic controls. OVA expressing MCA205 tumor cells were grown in 3D cultures for 48h in 21% O_2_ conditions. A subset of these cells were then moved to hypoxic (1% O2) conditions for an additional 24h. The hypoxic and normoxic spheroids were subsequently co-cultured with activated OT-I T cells. Tumor cells were identified by CellTracker staining (stained prior to co-culture) and cytotoxicity was assessed based on percent propidium idodide positive tumor cells. Each data point represents a replicate. Data is representative of 3 independent experiments. (**B**) Flow cytometry assessment of surface expression of MHC-SIINFEKL on OVA transfected MCA-205 cells. The tumor cells were grown as 3D spheroids for 48h in either 1% or 21% O_2_ conditions. Each data point represents an independent experiment. n = 4. Error bars indicate SD. The significance of differences was analyzed by the Student’s t-test (two-sided); *p = 0.02, ** p = 0.003 (A), p = 0.005 (B).

To determine whether reduced MHC-I expression correlated with poor antigen presentation to T cells, MCA205 sarcomas transfected with ovalbumin were used as targets in an *in vitro* cytotoxicity assay with OVA-specific OT-1 T cells. MCA205-OVA 3D spheroids were grown under either 1% or 21% O_2_ condition for 48h. As expected, cells grown under 1% O_2_ conditions had significantly reduced SIINFEKL-MHC expression (Fig 3B). The spheroids were then dispersed into single cells and co-cultured with activated OT-1 T cells at 21% O_2_ for 4h (Fig 3A). We show that CTLs are significantly more effective at killing cognate tumor cells that are grown under higher oxygen tension. This data suggests that downregulation of MHC class I by the hypoxic TME can make tumor cells poorly recognizable by effector T cells.

### Hyperoxia upregulates MHC class I expression in vitro in both 2D and 3D cultures

**Fig 4:**
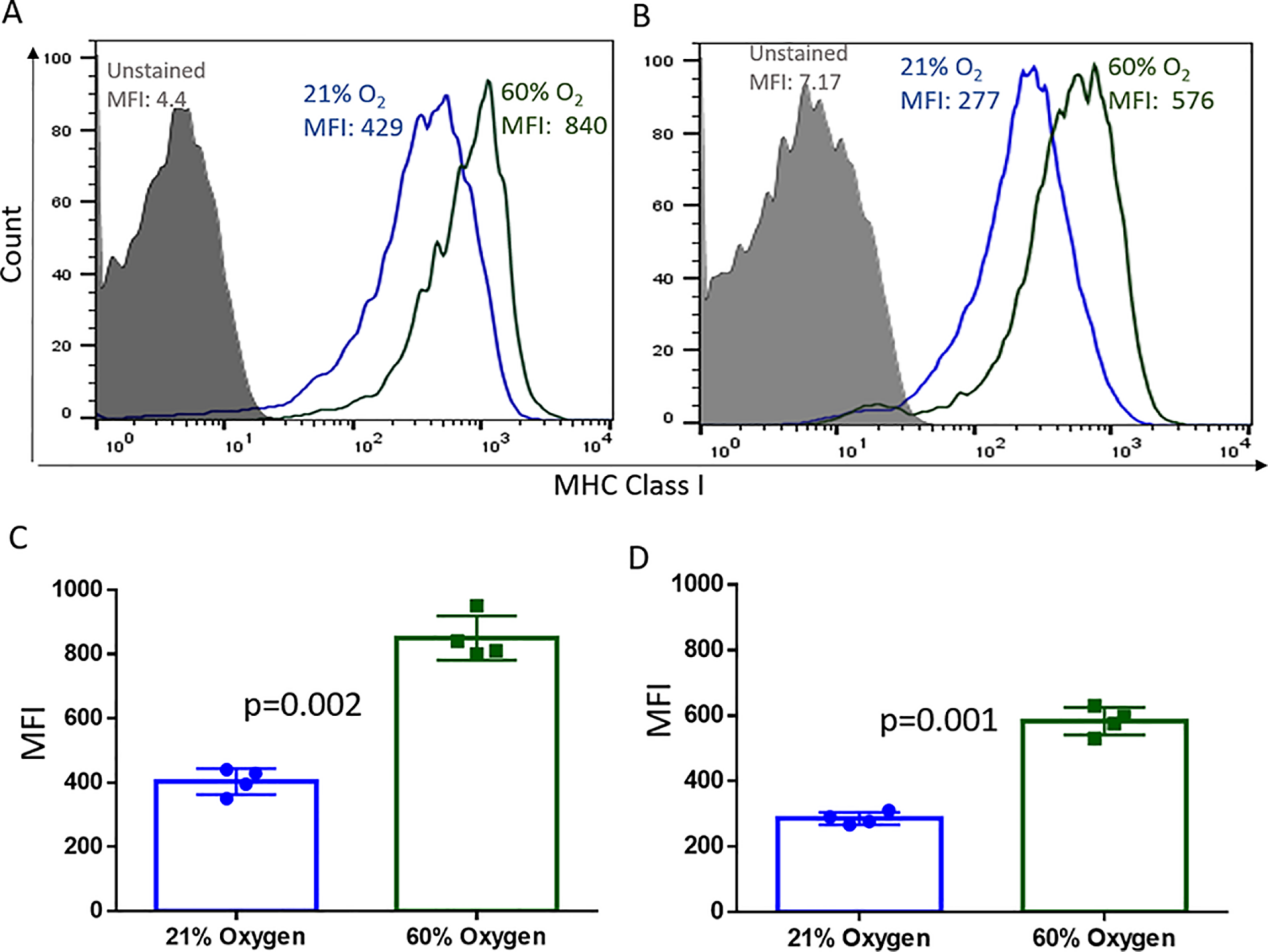
Hyperoxia upregulates MHC class I expression equally in 2D and 3D cultures. **(A-D):** MCA205 tumors were grown as 2D monolayers **(A, C)** or as 3D spheroids **(B, D)** at 21% O_2_ or 60% O_2_ for 48h. MHC class I levels were determined by flow cytometry. The magnitude of MHC class I upregulation was similar in 2D and 3D cultures. Representative histograms (**A, B**) and associated quantification and statistics (**C, D**) of 4 independent experiments shown. The significance of differences was analyzed by the Student’s t-test (two-sided); p = 0.002 (C), p = 0.001 (D). Grey filled: Unstained control; Blue: Normoxia (21% O_2_); Green: Hyperoxia (60% O_2_). Error bars indicate SD.

Validating oxygen-mediated regulation of MHC expression, exposure of tumor cells to hyperoxic atmosphere (60% oxygen) enhanced MHC class I expression (Fig 4A–4D; S3A–S3H Fig). This result is consistent with our previous report in which respiratory hyperoxia (60% oxygen) significantly increased MHC class I expression on tumor cells *in vivo* and enhanced clearance by anti-tumor T cells [31, 34]. The magnitude of MHC induction did not differ between 2D (Fig 4A and 4C) and 3D (Fig 4B and 4D) cultures.

### Molecular oxygen regulates MHC class I expression transcriptionally via HIF

**Fig 5:**
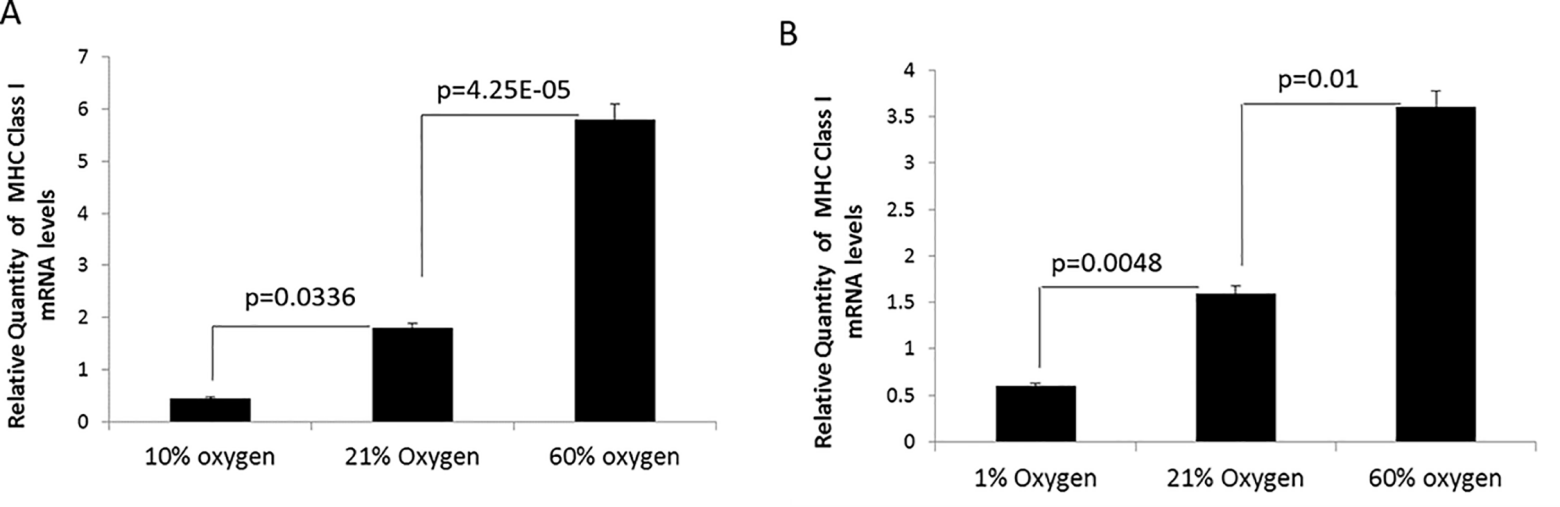
Molecular oxygen regulates MHC class I expression transcriptionally. **(A)** Mice bearing MCA205 pulmonary tumors were exposed to either respiratory hypoxia (10% O_2_), normoxia (21% O_2_), or respiratory hyperoxia (60% O_2_) for 48h. **(B)** MCA205 tumors grown *in vitro* in 3D spheroids under hypoxia (1% O_2_), normoxia (21% O_2_), or hyperoxia (60% O_2_) for 48h. Hypoxia significantly downregulated whereas hyperoxia significantly upregulated MHC class I transcripts as compared normoxic controls both *in vivo* and *in vitro*. RT-qPCR was used to analyze MHC class I (H-2Kb) transcript levels. Ribosomal protein L32 was used as internal control. Y- axis represents transcript levels relative to normoxic controls. n = 4. The significance of differences was analyzed by the Student’s t-test (two-sided); p values are as indicated in the figure. Error bars indicate SD.

Equipped with the appropriate *in vitro* platform, we next explored the mechanisms by which oxygen tension affected MHC expression. Using MCA205 spheroids cultured *in vitro* under 1%, 21%, or 60% O_2_ for 48h and MCA205 pulmonary tumors from mice exposed to 10%, 21%, or 60% O_2_ for 48h, MHC class I mRNA levels were analyzed by RT-qPCR. We found that hypoxia downregulated while hyperoxia upregulated MHC class I mRNA expression both *in vivo* (Fig 5A) and *in vitro* (Fig 5B).

**Fig 6:**
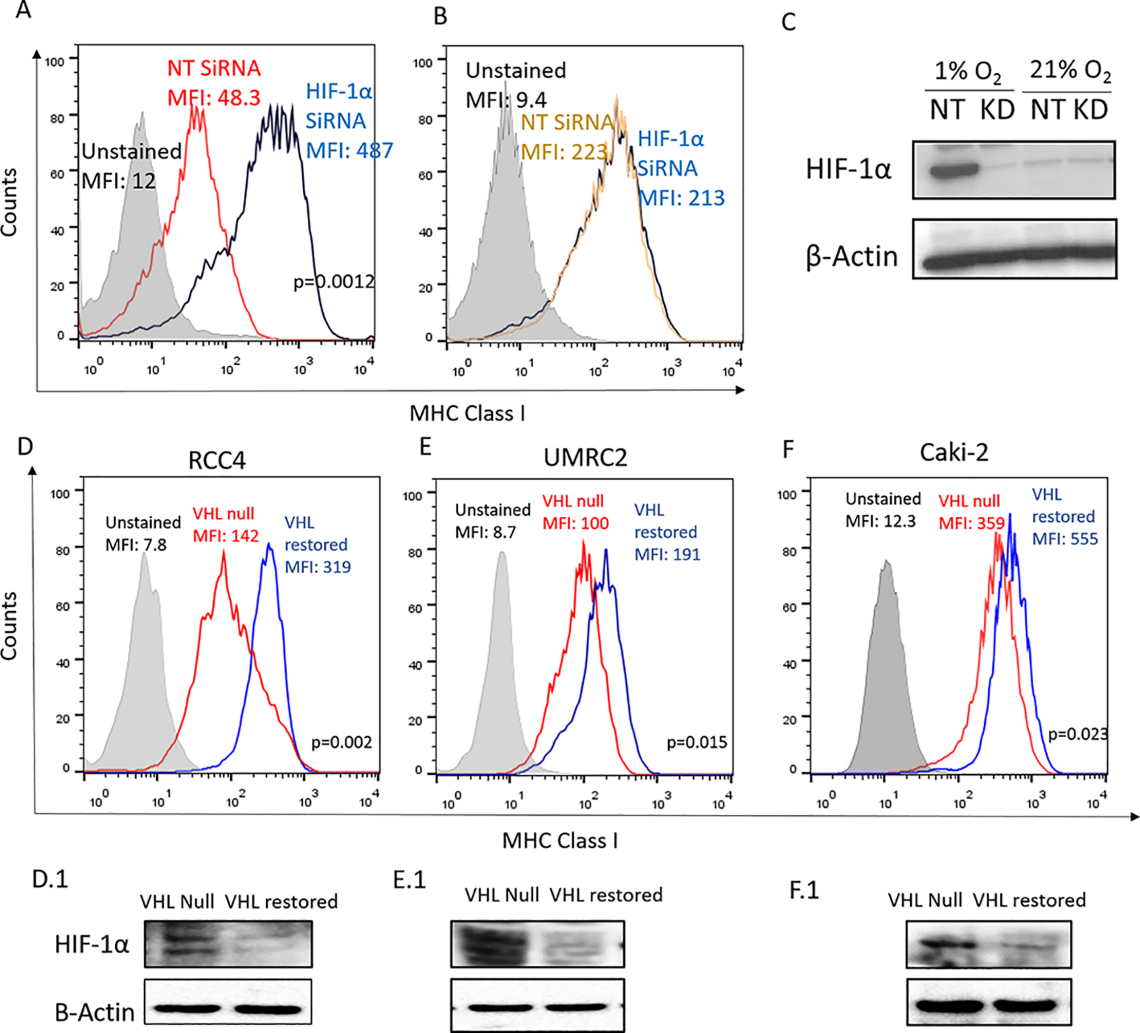
Hypoxia downregulates MHC class I expression via HIF transcription factors. **(A-C):** siRNA mediated knockdown of HIF-1α reversed hypoxic downregulation of MHC class I expression as compared with the scrambled, non-targeting (NT) siRNA control. MCA205 tumor cells were reverse transfected with scrambled siRNA (NT; red histogram) or with HIF-1α specific siRNA (blue histogram) and cultured as 3D spheroids under 1% **(A)** or 21% **(B)** oxygen for 48h. Levels of MHC class I surface expression was determined using flow cytometry. Efficacy of gene knockdown was assessed using Western blot **(C)**. β-Actin was used as the loading control. Representative data of 3 independent experiments shown. **(D-F):** Flow cytometry assessment of surface expression of HLA-ABC on paired isogenic renal cell carcinoma cell lines RCC4 **(D)**, UMRC2 **(E)** and CAKI2 **(F)**. Each pair had the parental cell line that lacked endogenous wild-type VHL (VHL null, transfected with empty vector) and one with vector stably expressing functional VHL (VHL restored). Restoring VHL function and thereby reducing HIF expression, significantly increased HLA-ABC expression on the cells. Representative histograms of 4 independent experiments are shown. Grey filled: unstained control; red: VHL null genotype; blue: VHL restored genotype. **(D1-F1):** Inactivation of HIF-1α by restoring VHL expression was verified by Western blotting for RCC4 **(D1)**, UMRC2 **(E1)** and CAKI2 **(F1)** cells. β-Actin was used as the loading control.

We next set out to determine if hypoxia affected MHC expression by HIF stabilization as it is known to be a critical transcriptional regulator of cells in hypoxic conditions [35]. siRNA-mediated knockdown of HIF-1α prevented hypoxia-mediated downregulation of both surface expression (Fig 6A; S4A and S5A Figs) and transcripts of MHC class I (S4B and S5C Figs). HIF-1α knockdown did not affect MHC expression in cells cultured under normoxic conditions (Fig 6B; S4A, S4B and S5B Figs). In 3D cultures at 1% O_2_, HIF-1α siRNA achieved greater than 90% knockdown of the protein as confirmed by Western blots (Fig 6C; S5D Fig). HIF-1α expression was not detectable in 21% O_2_ conditions. siRNA-mediated knockdown of HIF-2α also prevented hypoxia-mediated downregulation of both surface expression (S6A Fig) and transcripts of MHC class I (S6B Fig). Thus, HIF-1α and 2α may have redundant roles in regulating MHC class I expression.

To further confirm the role of HIF in MHC class I regulation, we used human renal cell carcinoma (RCC) cell lines that are extensively characterized and are known to have constitutively activated HIF expression due to inactivation of VHL gene [36]. We compared expression levels of MHC class I in paired isogenic cell lines transfected with empty vector (VHL null; HIF high) or vector expressing wild-type VHL (VHL restored; HIF low). These genetic control assays further validated our previous observations, since restoring VHL function, and thereby reducing HIF-1α levels (Fig 6D1–6F1), significantly upregulated MHC class I surface expression (Fig 6D–6F, S4C Fig) and transcript levels (S4D Fig). To corroborate the redundant role of HIF-2α in regulating MHC expression, isogenically paired RCCs that are known to express only the HIF-2α isoform were used [37]. Restoring VHL and thereby downregulating HIF-2α levels significantly upregulated both MHC class I surface expression (S6D and S6E Fig) and transcript levels (S6F Fig).

### Molecular oxygen affects expression of TAPs and LMPs

We next explored the effects of oxygen tension on constitutive expression of TAP1, TAP2, LMP2 and LMP7 proteins, both *in vivo* and *in vitro*. These components of the antigen processing machinery have a central role in determining the surface density of peptide-MHC complexes [27, 28] and have been shown to be transcriptionally downregulated by VHL/HIF *in vitro* [9].

**Fig 7:**
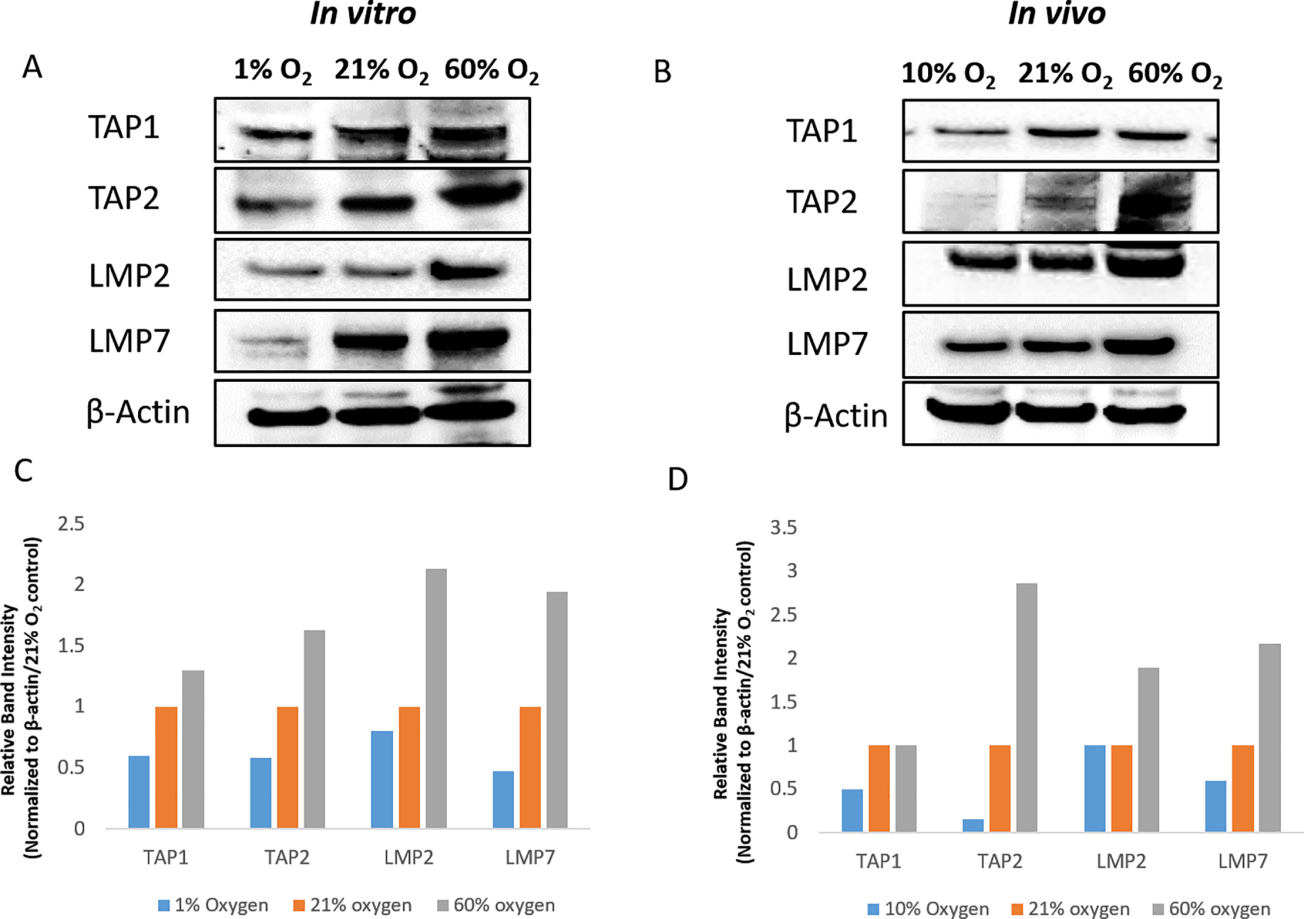
Hypoxia downregulates and hyperoxia upregulates expression levels of TAPs and LMPs. **(A, C)** MCA205 tumor cells were cultured *in vitro* as 3D spheroids for 48h under 1%, 21% or 60% oxygen. **(C)** Relative band intensity, normalized to the loading control and 21% oxygen samples is shown. **(B, D)** For *in vivo* experiments, tumor nodules (MCA205 pulmonary tumors) were harvested from mice exposed to respiratory hypoxia (10% oxygen), normoxia (21% oxygen) or hyperoxia (60% oxygen) for 48h. **(D)** Relative band intensity, normalized to the loading control and 21% oxygen samples is shown. Protein levels were determined by Western blot. β-actin was used as loading control. Representative blots with samples from 2 independent experiments are shown.

MCA205 pulmonary tumors from mice exposed to 10%, 21% or 60% O_2_ for 48h and MCA205 spheroids cultured *in vitro* under 1%, 21% or 60% O_2_ for 48h were analyzed by Western blot. Hypoxia significantly downregulated TAP1, TAP2 and LMP7 both *in vitro* (Fig 7A and 7C) and *in vivo* (Fig 7B and 7D), with modest effects on LMP2 expression. Hyperoxia upregulated expression of TAP2, LMP2 and LMP7 both *in vitro* (Fig 7A and 7C) and *in vivo* (Fig 7B and 7D), with no effect on TAP1 expression.

Positive correlation between oxygen tension and expression of genes critical to antigen presentation suggests that oxygen tension may dictate the density of cell surface peptide-MHC class I complexes. In agreement with this proposition, hyperoxia significantly upregulated MHC class I presentation of the immunodominant ovalbumin peptide SIINFEKL [38] in B16 melanoma cells and MCA-205 fibrosarcoma cells transfected with ovalbumin (S6A and S6B Fig).

## Discussion

Cancer cells with reduced or lost MHC expression avoid immunosurveillance and are not sufficiently immunogenic to activate anti-tumor immune responses [21]. Preventing MHC downregulation can potentiate the efficacy of many forms of cancer immunotherapy including cancer vaccines, adoptive cell transfer and immune checkpoint inhibitors [18, 39]. Expression of MHC is controlled by complex regulatory signals and can involve distinct transcriptional machinery under different microenvironments [23]. In this study, we provide evidence for oxygen tension to serve as an important extrinsic signal that regulates levels of MHC class I on tumors. Indeed, tissue hypoxia, which is frequently found in tumors [40], is shown to downregulate MHC class I expression.

Mechanistically, we show that oxygen tension affects transcription of MHC class I heavy chains, with hypoxia downregulating and hyperoxia upregulating mRNA levels of MHC class I (Fig 5). Further analysis revealed the role of HIF in regulating MHC expression (Fig 6). Data from studies using paired isogenic renal cell carcinomas with non-functional (VHL null; HIF-1α/2α^high^) and functional VHL protein (VHL restored; HIF-1α/2α^low^), along with siRNA of HIF, allowed us to firmly implicate the role of HIF in downregulating MHC class I expression. Interestingly, there is a noticeable delay from the induction of HIF and the downregulation of MHC class I. This observation, in conjunction with (i) an undefined hypoxia-response element in the MHC promoter (ii) evidence in literature that HIF-dependent gene suppression is typically due to indirect mechanisms rather than direct promoter binding [41], is indicative of a second regulator downstream of HIF. It remains to be established whether HIF-1α targets that are known to regulate MHC expression such as Signal Transducer and Activator of Transcription 1 (STAT1) and Interferon Regulatory Factor (IRF) 1 and 9 [9, 42] are involved.

Our data also attracts attention to the hyperoxia-mediated upregulation of MHC class I expression *in vivo* and *in vitro*. Since exposure to hyperoxic atmospheres can reverse tissue hypoxia and HIF-1α expression in tumors [31], hyperoxia may increase MHC class I levels by blocking the hypoxia-mediated MHC downregulation. However, the *in vitro* data suggests the presence of a second mechanism. *In vitro* cell culture at 21% oxygen, especially as conventional 2D cultures, is not deeply hypoxic (S2 Fig, Fig 6C), but MHC expression was significantly upregulated by 60% oxygen. This result suggests a mechanism independent of HIF degradation. Our working hypothesis for this *in vitro* observation is that molecular oxygen is able to recruit an otherwise latent transcriptional network independent of hypoxia and HIF, which provides a synergistic effect on overall MHC class I expression. In addition, previous studies have shown that the core promoter of MHC class I gene is comparable to a ‘flexible platform’ that recruits different transcription factors based on microenvironmental cues [24, 43]. Future work will determine if reactive oxygen species, which have been shown to behave as signaling molecules [44, 45], have a role in hyperoxic upregulation of MHC expression *in vitro*.

In addition to transcriptional regulation of MHC, we sought to study the effects of oxygen tension on TAPs and LMPs since these components of the antigen processing pathway have been shown to have a dominant role in determining the density of cell surface peptide-MHC complexes [26, 28] and were identified as potential targets of the VHL and HIF-1α pathway [9]. We found that hypoxia downregulated TAP1, TAP2 and LMP7 both *in vitro* and *in vivo*, while hyperoxia significantly upregulated TAP2, LMP2 and LMP7 (Fig 7). These results suggest that oxygen regulates MHC-peptide presentation not only transcriptionally but also through the modulation of peptide generation and transport into the endoplasmic reticulum.

Taken together, these data firmly suggests that oxygen tension has a key role in determining tumor immunogenicity. Further support for this study comes from our previous observations [31] of lower tumoral MHC class I expression in hypoxic regions of the TME and studies in renal cell carcinomas which showed VHL mutations (i) conferred increased susceptibility to natural killer cell-mediated lysis [46] and (ii) identified several genes of the antigen-presentation pathway, including MHC class I and II genes, as potential targets of VHL in a RNA screen of interferon-stimulated responsive element (IRSE) regulated genes [9]. Thus, the current study provides a justification for the therapeutic oxygenation or inhibition of HIF-α-mediated signaling in combination with immunotherapies of cancer [31, 34]. Indeed, reversal of hypoxia by oxygen supplementation may deprive tumors of yet another strategy to evade the immune response at the level of antigen recognition. From a translational point of view, the hyperoxia-induced upregulation of MHC expression *in vitro* has important implications for improving efficacy of tumor-cell based vaccines by increasing the surface density of peptide-MHC molecules. In addition, culturing tumor cells under hyperoxic conditions may also be beneficial in discovering new immune epitopes that were perhaps below the limit of detection previously.

Our study also highlights the importance of choosing the right *in vitro* platform in investigating complex biological interactions. Mechanistic understanding of hypoxia-mediated MHC downregulation described here would remain indefinable without overcoming the limitations of routinely-used 2D *in vitro* cell culture systems. Of methodological significance, these data also offer a proof of principle for a novel approach to reproduce the physiologically relevant biological effects of hypoxic exposure by switching to 3D culture.

In conclusion, we have provided *in vivo* and *in vitro* evidence that the TME mediated hypoxia-HIF-α signaling cascade decreases expression of MHC class I and could provide tumors with a fundamental mechanism to evade immunosurveillance by CTLs.

## Materials and methods

### Cell lines

MCA205 (gift from Dr. Suyu Shu, Cleveland clinic), 4T.1 (ATCC #CRL-2539), P815 (ATCC #TIB-64), RMA (gift from Dr. Scott Abrams, NCI), B16 transfected with ovalbumin (gift from Dr. Kai W. Wucherpfennig, DFCI), MCA205 transfected with ovalbumin (gift from Dr. John M. Routes, Children's Hospital of Wisconsin), EL4 (ATCC #TIB-39), renal cell carcinoma cells RCC4, UMRC2, Caki-2, A-498 and 786–0 with or without the lentiviral vector (pcDNA3) for VHL gene (gift from Dr. William G. Kaelin, DFCI [47]), were cultured in RPMI-1640, 10% FBS.

### 2D and 3D culture systems

Cells were cultured as monolayers using conventional 96-well or 35mm culture plates (Corning; # CLS3603; #430165). For spheroid culture, cells were grown in Scivax plates, either the 35mm dishes or 96-well plates (Scivax; #NCD-LS35-5; #NCP-LS96). Cells were cultured for 24-48h to allow for the formation of spheroids before using in experiments, with the exception of RNAi experiments. Images of cells in 2D and 3D systems were captured using an inverted microscope fitted with Olympus camera.

### Animals

Female C57BL/6J (B6) mice, 11 weeks old, were purchased from Jackson Laboratory. All animal experiments were conducted in accordance with IACUC guidelines of Northeastern University (protocol approved by IACUC of Northeastern university. Protocol number: 13–0928 R, previous number: 10–1031 R).

### Tumors

For establishment of pulmonary tumors, B6 mice were injected intravenously with 1×10^5^ MCA205 cells suspended in 200μl of HBSS. For establishment of subcutaneous tumors, B6 mice were injected in the flank with 3×10^5^ MCA205 cells suspended in 100μl of HBSS. When tumors reached 200-250mm^3^, day 11 (pulmonary tumors) or day 15 (subcutaneous tumors), mice were randomly divided into three groups and exposed to normoxia (21% O_2_), hypoxia (10% O_2_) or hyperoxia (60% O_2_) for 48h. Hypoxic exposure was achieved using the Biospherix Oxycycler (#A84XOV). Hyperoxic (60% oxygen) exposure was achieved using chambers as described previously [34]. Mice were monitored for health status daily and tumor volumes were monitored thrice a week using a digital calipers. Post oxygen treatments, mice were humanely euthanized by carbon dioxide inhalation and tumors collected for further analysis. No anesthetics or analgesics were used.

### *In vitro* hypoxic and hyperoxic culture and Hypoxyprobe analysis

Cells grown in 2D and 3D culture systems were exposed to hypoxia (1% O_2_, 5% CO_2_), normoxia (21% O_2_, 5% CO_2_) or hyperoxia (60% O_2_, 5% CO_2_) for 48h. Hypoxyprobe™-1 Kit (Hypoxyprobe Inc; #HP1-200Kit) was used to detect hypoxia. Briefly, cells in 2D and 3D systems were incubated with 150 μM of Hypoxyprobe for 1h. The cells were then extensively washed and levels of MHC class I and Hypoxyprobe were assessed using flow cytometry.

### Flow cytometry

Pulmonary or solid tumors were excised and subjected to gentle mechanical dissociation by chopping the tumors into small sections and teasing apart tissue with needles. Enzymatic dissociation of tumors was intentionally avoided to prevent damage to cell surface markers. Cells cultured in 2D cultures were harvested by using cell dissociation buffer (Thermo Scientific; #13151–014). 3D spheroids were harvested and dissociated by gentle pipetting. The cells were then incubated with mAbs, and acquired on a FACSCalibur. Monoclonal antibodies used were mouse MHC class I (Biolegend; #116518), mouse H-2K^b^-SIINFEKL (Biolegend; #141603), human HLA-ABC (eBiosciences; #11-9983-42), Hypoxyprobe (Hypoxyprobe Inc; #HP1-200Kit). Dead cells were excluded by using the live/dead fixable dead cell stain (Invitrogen, #L34961). Tumor cells were analyzed using FlowJo (Tree Star) software.

### Reverse transcription polymerase chain reaction (RT-PCR)

RNA was extracted from mice with 11-day established pulmonary tumors or spheroids treated with different oxygen tensions for 48h using the RNeasy Kit (Qiagen; #74104). RT-PCR was performed using the RT2 SYBR green PCR master mix (Life Technologies, #4309155) on an Applied Biosciences 7300. Primers were purchased from SABiosciences (Qiagen; #PPM25849D-200). Ribosomal protein L32 (Qiagen; #PPM03300B-200) was included as the internal control.

### RNA interference

MCA205 cells were reverse transfected with scrambled control (Dharmacon; #D-001810-10) or siRNA specific to HIF-1α gene (Dharmacon; #L-040638-00-0005) or HIF-2α gene (Dharmacon; #L-040635-01-0005), using Lipofectamine reagent per manufacturer’s instructions (Thermo; #13778030). The cells were then grown on Scivax plates for 48h under 21% or 1% O_2_. Efficacy of gene knockdown was assessed using Western blot.

### Western blot

Spheroids and *in vivo* tumor samples were lysed in RIPA buffer (Boston Bioproducts; #BP-115) with protease and phosphatase inhibitors (AG Scientific; #P-1551). 40μg of protein was loaded per sample and fractionation with SDS–PAGE was followed by wet transfer to PVDF membrane. Antibodies used were HIF-1α (Novus Biologicals; #NB100-479); HIF-2α (CST; #7096); TAP1 (Santa Cruz; #sc-11465); TAP2 (Santa Cruz; #sc-25612); LMP2 (Santa Cruz; #sc-373689); LMP7 (Cell Signaling; #D1K7X); β-actin (Sigma-Aldrich; #A2228).

### T cell stimulation and cytotoxicity assay

Splenocytes from OT-1 mice were activated *in vitro* with 100 ug/mL OVA for 48h. CD8 T cells were then isolated (R&D; #MAGM203) and used in the cytotoxicity assay.

Ovalbumin-transfected MCA205 tumor cells were stained with Cell Tracker Deep Red (Thermofisher; #C34565) and grown in 3D cultures for 24h in 21% or 1% O_2_ post-spheroid formation. Spheroids were dissociated using the spheroid dispersion solution (Scivax; #SD4X) before use in cytotoxicity assays. To assess cytotoxicity, 10,000 target cells were incubated for 4h at 37°C, 21% O_2_, with different ratios of effector T cells as indicated. At the end of 4h, propidium iodide was used to determine the proportion of dead tumor cells (cell tracker +, PI+).

### Statistics

The significance of differences was analyzed by the Student’s t-test (two-sided). *P* values are listed within the figures and figure legends.

## Supporting information

S1 Fig Hypoxia downregulates MHC class I expression *in vitro* in 3-dimensional (3D) but not in 2-dimensional (2D) culture systems. 4T.1 breast carcinoma **(S1 A,B)**, P815 mastocytoma **(S1 C,D)**, RMA T lymphoma **(S1 E,F)** and EL4 thymoma **(S1 G,H)** were cultured as 2D monolayers (indicated as 2D) or as 3D spheroids (indicated as 3D) and cultured under 21% O_2_ or 1% O_2_ for 48h. Levels of MHC class I expression was determined using flow cytometry. Representative histograms of 4 independent experiments are shown. Grey filled: unstained control; red: hypoxia; blue: normoxia. MFI: mean fluorescence intensity. Inset of S1 G,H: 40X magnification of El4 cells grown at 1% O_2_ in 2D culture **(G)** or in 3D culture **(H)** representing the growth of these cells in clumps irrespective of the culture format.
(TIF)

S2 Fig Detection of hypoxic regions in 3D spheroids cultured under normoxic conditions. MCA-205 fibrosarcoma cells were grown as spheroids **(S2A)** or flat monolayer **(S2B)** in normoxic conditions (21% oxygen) and levels of hypoxia in each culture system assessed using hypoxyprobe. About 16% of the population was hypoxic in the 3D spheroids whereas there was no detectable levels of hypoxia in the 2D cultured cells. In 3D spheroids of EL4, about 20% of the population was hypoxic **(S2D)**. Representative contour plots of three independent experiments shown. **(S2C, E)** Mean fluorescent intensity (MFI) of MHC class I expression on 3D spheroids (MCA205; S2C, EL4; S2E) from less hypoxic (HP low) and more hypoxic (HP high) regions showed inverse correlation between hypoxia and MHC class I expression. Each point on graph represents an independent experiment with the average represented as a dash.
(TIF)

S3 Fig Hyperoxia upregulates MHC class I expression equally in 2D and 3D cultures. 4T.1 breast carcinoma **(S3 A,B)**, P815 mastocytoma **(S3 C,D)**, RMA T lymphoma **(S3 E,F)** and EL4 thymoma **(S3 G,H)** were cultured as 2D monolayers (indicated as 2D) or as 3D spheroids (indicated as 3D) and cultured under 21% O_2_ or 60% O_2_ for 48h. Levels of MHC class I expression was determined using flow cytometry. Representative histograms of 4 independent experiments are shown. Grey filled: unstained control; blue: normoxia; green: hyperoxia. MFI: mean fluorescence intensity.
(TIF)

S4 Fig Hypoxia downregulates MHC class I expression via HIF transcription factors; extended data from Fig 6. **(SA, B):** siRNA mediated knockdown of HIF-1α reversed hypoxic downregulation of MHC class I expression as compared with the scrambled, non-targeting (NT) siRNA control. MCA205 tumor cells were reverse transfected with scrambled siRNA (NT) or with HIF-1α specific siRNA and cultured as 3D spheroids under 1% or 21% oxygen for 48h. Levels of MHC class I surface expression was determined using flow cytometry; quantitative analysis of representative histograms shown in Fig 6 shown here **(A)**. Levels of MHC class I transcripts were assessed using RT-qPCR **(B)**. Average data of 3 independent experiments are shown.
**(S4C, D):** Flow cytometry assessment of surface expression of HLA-ABC (**S4C**) and RT-qPCR analysis of HLA-ABC transcript levels (**S4D**) on paired isogenic renal cell carcinoma cell lines RCC4, UMRC2 and CAKI2. Each pair had the parental cell line that lacked endogenous wild-type VHL (VHL null, transfected with empty vector) and one with vector stably expressing functional VHL (VHL restored). Restoring VHL function and thereby reducing HIF expression, significantly increased HLA-ABC surface expression and transcript levels in the cells. Average data of 4 independent experiments are shown.
(TIF)

S5 Fig Hypoxia downregulates MHC Class I expression via Hif-1α(S5A-D). siRNA mediated knockdown of HIF-1α reversed hypoxic downregulation of MHC class I expression as compared with the scrambled, non-targeting (NT) siRNA control. EL4 tumor cells were reverse transfected with scrambled siRNA (NT; Red histogram) or with HIF-1α specific siRNA (blue histogram) and cultured as 3D spheroids under 1% **(S5A)** or 21% **(S5B)** oxygen for 48h. Levels of MHC class I surface expression was determined using flow cytometry **(S5A,B)**. RT-qPCR was used to analyze MHC class I transcript levels. Ribosomal protein L32 was used as internal control **(S5C)**. Efficacy of gene knockdown was assessed using western blot **(S5D)**. β-actin was used as the loading control. Representative data of 2 independent experiments are shown.
(PNG)

S6 Fig HIF-1α and HIF-2α have redundant roles in downregulating MHC class I expression. **(S6A-C):** siRNA mediated knockdown of HIF-1α, HIF-2α or both reversed hypoxic downregulation of MHC class I expression as compared with the scrambled, non-targeting (NT) siRNA control. MCA205 tumor cells were reverse transfected with scrambled siRNA (NT) or with HIF-1α, HIF-2α or both HIF-1α and HIF-2α specific siRNA and cultured as 3D spheroids under 1% or 21% oxygen for 48h. Levels of MHC class I surface expression was determined using flow cytometry (**S6A**). Transcripts levels were determined by RT-qPCR, with Ribosomal protein L32 as internal control (**S6B**). Efficacy of gene knockdown was assessed using western blot **(S6C)**. Greater than 90% knockdown of HIF-1α/ HIF-2α was achieved. β-actin was used as the loading control. Average data of 3 independent experiments are shown.
**(S6D-F):** Flow cytometry assessment of surface expression of HLA-ABC on paired isogenic renal cell carcinoma cell lines A-498 **(D)** and 786–0 **(E)**. Each pair had the parental cell line that lacked endogenous wild-type VHL (VHL null, transfected with empty vector) and one with vector stably expressing functional VHL (VHL restored). Restoring VHL function and thereby reducing HIF expression, significantly increased HLA-ABC expression on the cells (**S6D,E**) and transcript levels (**S6F**). Representative histograms of 4 independent experiments are shown. Grey filled: Unstained control; Red: VHL null phenotype; Blue: VHL restored phenotype. For RT-qPCR, average data from 4 independent experiments shown.
(TIF)

S7 Fig Hyperoxia upregulates presentation of ovalbumin peptide SIINFEKL. (**S7A,B**) 60% O_2_ significantly upregulated expression of the immunodominant peptide of ovalbumin, SIINFEKL. B16 melanoma cells (**S7A**) or MCA205 fibrosarcoma cells (**S7B**) transfected with ovalbumin were cultured as 3D spheroids for 48h either under 21% or 60% O_2_. Levels of MHC class I-SIINFEKL surface expression was determined using flow cytometry, with overnight incubation at 4°C with the mAb to MHC-SIINFEKL. Representative histograms of 4 independent experiments are shown. Grey filled: unstained control; blue: normoxia; green: hyperoxia. MFI: mean fluorescent intensity.
(TIF)

## References

[1] Quail DF, Joyce JA. Microenvironmental regulation of tumor progression and metastasis. Nature medicine. 2013;19(11):1423–37. Epub 2013/11/10. doi: 10.1038/nm.3394 ; PubMed Central PMCID: PMCPmc3954707. 24202395 10.1038/nm.3394 PMC3954707

[2] Gajewski TF, Schreiber H, Fu YX. Innate and adaptive immune cells in the tumor microenvironment. Nature immunology. 2013;14(10):1014–22. doi: 10.1038/ni.2703 ; PubMed Central PMCID: PMC4118725. 24048123 10.1038/ni.2703 PMC4118725

[3] Schmitt TM, Stromnes IM, Chapuis AG, Greenberg PD. New Strategies in Engineering T-cell Receptor Gene-Modified T Cells to More Effectively Target Malignancies. Clinical cancer research: an official journal of the American Association for Cancer Research. 2015 Epub 2015/10/16. doi: 10.1158/1078-0432.CCR-15-0860 . 26463711 10.1158/1078-0432.CCR-15-0860 PMC4746077

[4] Philip B, Ito K, Moreno-Sanchez R, Ralph SJ. HIF expression and the role of hypoxic microenvironments within primary tumours as protective sites driving cancer stem cell renewal and metastatic progression. Carcinogenesis. 2013;34(8):1699–707. doi: 10.1093/carcin/bgt209 . 23740838 10.1093/carcin/bgt209

[5] Siemens DR, Hu N, Sheikhi AK, Chung E, Frederiksen LJ, Pross H, et al Hypoxia increases tumor cell shedding of MHC class I chain-related molecule: role of nitric oxide. Cancer research. 2008;68(12):4746–53. doi: 10.1158/0008-5472.CAN-08-0054 . 18559521 10.1158/0008-5472.CAN-08-0054

[6] Eltzschig HK, Sitkovsky MV, Robson SC. Purinergic signaling during inflammation. The New England journal of medicine. 2012;367(24):2322–33. Epub 2012/12/14. doi: 10.1056/NEJMra1205750 ; PubMed Central PMCID: PMCPmc3675791. 23234515 10.1056/NEJMra1205750 PMC3675791

[7] Barsoum IB, Koti M, Siemens DR, Graham CH. Mechanisms of hypoxia-mediated immune escape in cancer. Cancer research. 2014;74(24):7185–90. Epub 2014/10/26. doi: 10.1158/0008-5472.CAN-14-2598 . 25344227 10.1158/0008-5472.CAN-14-2598

[8] Blankenstein T, Coulie PG, Gilboa E, Jaffee EM. The determinants of tumour immunogenicity. Nature reviews Cancer. 2012;12(4):307–13. doi: 10.1038/nrc3246 ; PubMed Central PMCID: PMC3552609. 22378190 10.1038/nrc3246 PMC3552609

[9] Ivanov SV, Salnikow K, Ivanova AV, Bai L, Lerman MI. Hypoxic repression of STAT1 and its downstream genes by a pVHL/HIF-1 target DEC1/STRA13. Oncogene. 2007;26(6):802–12. doi: 10.1038/sj.onc.1209842 . 16878149 10.1038/sj.onc.1209842

[10] Srivastava PK, Duan F. Harnessing the antigenic fingerprint of each individual cancer for immunotherapy of human cancer: genomics shows a new way and its challenges. Cancer immunology, immunotherapy: CII. 2013;62(5):967–74. Epub 2013/04/23. doi: 10.1007/s00262-013-1422-x ; PubMed Central PMCID: PMCPmc3634982. 23604106 10.1007/s00262-013-1422-x PMC3634982

[11] Pradeu T, Carosella ED. On the definition of a criterion of immunogenicity. Proceedings of the National Academy of Sciences of the United States of America. 2006;103(47):17858–61. doi: 10.1073/pnas.0608683103 ; PubMed Central PMCID: PMC1693837. 17101995 10.1073/pnas.0608683103 PMC1693837

[12] Smyth MJ, Dunn GP, Schreiber RD. Cancer immunosurveillance and immunoediting: the roles of immunity in suppressing tumor development and shaping tumor immunogenicity. Advances in immunology. 2006;90:1–50. Epub 2006/05/30. doi: 10.1016/S0065-2776(06)90001-7 . 16730260 10.1016/S0065-2776(06)90001-7

[13] Matsushita H, Vesely MD, Koboldt DC, Rickert CG, Uppaluri R, Magrini VJ, et al Cancer exome analysis reveals a T-cell-dependent mechanism of cancer immunoediting. Nature. 2012;482(7385):400–4. Epub 2012/02/10. doi: 10.1038/nature10755 ; PubMed Central PMCID: PMCPmc3874809. 22318521 10.1038/nature10755 PMC3874809

[14] Grandis JR, Falkner DM, Melhem MF, Gooding WE, Drenning SD, Morel PA. Human leukocyte antigen class I allelic and haplotype loss in squamous cell carcinoma of the head and neck: clinical and immunogenetic consequences. Clinical cancer research: an official journal of the American Association for Cancer Research. 2000;6(7):2794–802. Epub 2000/07/29. PubMed . 10914726

[15] Seliger B, Wollscheid U, Momburg F, Blankenstein T, Huber C. Characterization of the major histocompatibility complex class I deficiencies in B16 melanoma cells. Cancer research. 2001;61(3):1095–9. Epub 2001/02/28. PubMed . 11221838

[16] Garrido C, Romero I, Berruguilla E, Cancela B, Algarra I, Collado A, et al Immunotherapy eradicates metastases with reversible defects in MHC class I expression. Cancer Immunology, Immunotherapy. 2011;60(9):1257–68. doi: 10.1007/s00262-011-1027-1 21553283 10.1007/s00262-011-1027-1 PMC11028956

[17] Meissner M, Reichert TE, Kunkel M, Gooding W, Whiteside TL, Ferrone S, et al Defects in the human leukocyte antigen class I antigen processing machinery in head and neck squamous cell carcinoma: association with clinical outcome. Clinical cancer research: an official journal of the American Association for Cancer Research. 2005;11(7):2552–60. Epub 2005/04/09. doi: 10.1158/1078-0432.CCR-04-2146 . 15814633 10.1158/1078-0432.CCR-04-2146

[18] Aptsiauri N, Carretero R, Garcia-Lora A, Real LM, Cabrera T, Garrido F. Regressing and progressing metastatic lesions: resistance to immunotherapy is predetermined by irreversible HLA class I antigen alterations. Cancer immunology, immunotherapy: CII. 2008;57(11):1727–33. Epub 2008/05/21. doi: 10.1007/s00262-008-0532-3 . 18491093 10.1007/s00262-008-0532-3 PMC11030993

[19] Carretero R, Romero JM, Ruiz-Cabello F, Maleno I, Rodriguez F, Camacho FM, et al Analysis of HLA class I expression in progressing and regressing metastatic melanoma lesions after immunotherapy. Immunogenetics. 2008;60(8):439–47. doi: 10.1007/s00251-008-0303-5 . 18545995 10.1007/s00251-008-0303-5

[20] Reits EA, Hodge JW, Herberts CA, Groothuis TA, Chakraborty M, Wansley EK, et al Radiation modulates the peptide repertoire, enhances MHC class I expression, and induces successful antitumor immunotherapy. The Journal of experimental medicine. 2006;203(5):1259–71. Epub 2006/04/26. doi: 10.1084/jem.20052494 ; PubMed Central PMCID: PMCPmc3212727. 16636135 10.1084/jem.20052494 PMC3212727

[21] Escors D. Tumour immunogenicity, antigen presentation and immunological barriers in cancer immunotherapy. New journal of science. 2014;2014 Epub 2014/03/19. doi: 10.1155/2014/734515 ; PubMed Central PMCID: PMCPmc3952940. 24634791 10.1155/2014/734515 PMC3952940

[22] van den Elsen PJ, Holling TM, Kuipers HF, van der Stoep N. Transcriptional regulation of antigen presentation. Current opinion in immunology. 2004;16(1):67–75. Epub 2004/01/22. PubMed . 14734112 10.1016/j.coi.2003.11.015

[23] Howcroft TK, Raval A, Weissman JD, Gegonne A, Singer DS. Distinct transcriptional pathways regulate basal and activated major histocompatibility complex class I expression. Molecular and cellular biology. 2003;23(10):3377–91. Epub 2003/05/02. PubMed doi: 10.1128/MCB.23.10.3377-3391.2003 ; PubMed Central PMCID: PMCPmc154244. 12724398 10.1128/MCB.23.10.3377-3391.2003 PMC154244

[24] Mu J, Tai X, Iyer SS, Weissman JD, Singer A, Singer DS. Regulation of MHC class I expression by Foxp3 and its effect on regulatory T cell function. Journal of immunology. 2014;192(6):2892–903. Epub 2014/02/14. doi: 10.4049/jimmunol.1302847 ; PubMed Central PMCID: PMCPmc3952169. 24523508 10.4049/jimmunol.1302847 PMC3952169

[25] van den Elsen PJ. Expression regulation of major histocompatibility complex class I and class II encoding genes. Frontiers in immunology. 2011;2:48 Epub 2011/01/01. doi: 10.3389/fimmu.2011.00048 ; PubMed Central PMCID: PMCPmc3342053. 22566838 10.3389/fimmu.2011.00048 PMC3342053

[26] Oliveira CC, Querido B, Sluijter M, Derbinski J, van der Burg SH, van Hall T. Peptide transporter TAP mediates between competing antigen sources generating distinct surface MHC class I peptide repertoires. European journal of immunology. 2011;41(11):3114–24. Epub 2011/09/08. doi: 10.1002/eji.201141836 . 21898382 10.1002/eji.201141836

[27] Montoya M, Del Val M. Intracellular rate-limiting steps in MHC class I antigen processing. Journal of immunology. 1999;163(4):1914–22. Epub 1999/08/10. PubMed . 10438926

[28] Sijts AJ, Standera S, Toes RE, Ruppert T, Beekman NJ, van Veelen PA, et al MHC class I antigen processing of an adenovirus CTL epitope is linked to the levels of immunoproteasomes in infected cells. Journal of immunology. 2000;164(9):4500–6. Epub 2000/04/26. PubMed . 10779750 10.4049/jimmunol.164.9.4500

[29] Kaelin WG Jr. The von Hippel-Lindau tumour suppressor protein: O2 sensing and cancer. Nature reviews Cancer. 2008;8(11):865–73. Epub 2008/10/17. doi: 10.1038/nrc2502 . 18923434 10.1038/nrc2502

[30] Kaelin WG Jr. The von Hippel-Lindau tumor suppressor gene and kidney cancer. Clinical cancer research: an official journal of the American Association for Cancer Research. 2004;10(18 Pt 2):6290s–5s. Epub 2004/09/28. doi: 10.1158/1078-0432.CCR-sup-040025 . 15448019 10.1158/1078-0432.CCR-sup-040025

[31] Hatfield SM, Kjaergaard J, Lukashev D, Belikoff B, Schreiber TH, Sethumadhavan S, et al Systemic oxygenation weakens the hypoxia and hypoxia inducible factor 1alpha-dependent and extracellular adenosine-mediated tumor protection. Journal of molecular medicine. 2014;92(12):1283–92. Epub 2014/08/15. doi: 10.1007/s00109-014-1189-3 ; PubMed Central PMCID: PMCPmc4247798. 25120128 10.1007/s00109-014-1189-3 PMC4247798

[32] Page P, DeJong J. Effect of serum and oxygen concentration on gene expression and secretion of paracrine factors by mesenchymal stem cells. 2014;2014:601063 doi: 10.1155/2014/601063 . 25614742 10.1155/2014/601063 PMC4295344

[33] Ohta A, Diwanji R, Kini R, Subramanian M, Ohta A, Sitkovsky M. In vivo T cell activation in lymphoid tissues is inhibited in the oxygen-poor microenvironment. Frontiers in immunology. 2011;2:27 Epub 2011/01/01. doi: 10.3389/fimmu.2011.00027 ; PubMed Central PMCID: PMCPmc3342240. 22566817 10.3389/fimmu.2011.00027 PMC3342240

[34] Hatfield SM, Kjaergaard J, Lukashev D, Schreiber TH, Belikoff B, Abbott R, et al Immunological mechanisms of the antitumor effects of supplemental oxygenation. Science translational medicine. 2015;7(277):277ra30 Epub 2015/03/06. doi: 10.1126/scitranslmed.aaa1260 . 25739764 10.1126/scitranslmed.aaa1260 PMC4641038

[35] Semenza GL. HIF-1 mediates metabolic responses to intratumoral hypoxia and oncogenic mutations. The Journal of clinical investigation. 2013;123(9):3664–71. Epub 2013/09/04. doi: 10.1172/JCI67230 ; PubMed Central PMCID: PMCPmc3754249. 23999440 10.1172/JCI67230 PMC3754249

[36] Shen C, Kaelin WG Jr. The VHL/HIF axis in clear cell renal carcinoma. Seminars in cancer biology. 2013;23(1):18–25. Epub 2012/06/19. doi: 10.1016/j.semcancer.2012.06.001 ; PubMed Central PMCID: PMCPmc3663044. 22705278 10.1016/j.semcancer.2012.06.001 PMC3663044

[37] Shinojima T, Oya M, Takayanagi A, Mizuno R, Shimizu N, Murai M. Renal cancer cells lacking hypoxia inducible factor (HIF)-1alpha expression maintain vascular endothelial growth factor expression through HIF-2alpha. Carcinogenesis. 2007;28(3):529–36. Epub 2006/08/22. doi: 10.1093/carcin/bgl143 . 16920734 10.1093/carcin/bgl143

[38] Cascio P, Hilton C, Kisselev AF, Rock KL, Goldberg AL. 26S proteasomes and immunoproteasomes produce mainly N-extended versions of an antigenic peptide. The EMBO journal. 2001;20(10):2357–66. Epub 2001/05/15. doi: 10.1093/emboj/20.10.2357 ; PubMed Central PMCID: PMCPmc125470. 11350924 10.1093/emboj/20.10.2357 PMC125470

[39] White CA, Thomson SA, Cooper L, van Endert PM, Tampe R, Coupar B, et al Constitutive transduction of peptide transporter and HLA genes restores antigen processing function and cytotoxic T cell-mediated immune recognition of human melanoma cells. International journal of cancer Journal international du cancer. 1998;75(4):590–5. Epub 1998/02/18. PubMed . 9466661 10.1002/(sici)1097-0215(19980209)75:4<590::aid-ijc16>3.0.co;2-d

[40] Leo C, Giaccia AJ, Denko NC. The hypoxic tumor microenvironment and gene expression. Seminars in radiation oncology. 2004;14(3):207–14. Epub 2004/07/16. doi: 10.1016/j.semradonc.2004.04.007 . 15254863 10.1016/j.semradonc.2004.04.007

[41] Mole DR, Blancher C, Copley RR, Pollard PJ, Gleadle JM, Ragoussis J, et al Genome-wide association of hypoxia-inducible factor (HIF)-1alpha and HIF-2alpha DNA binding with expression profiling of hypoxia-inducible transcripts. The Journal of biological chemistry. 2009;284(25):16767–75. Epub 2009/04/24. doi: 10.1074/jbc.M901790200 ; PubMed Central PMCID: PMCPmc2719312. 19386601 10.1074/jbc.M901790200 PMC2719312

[42] Jarosinski KW, Massa PT. Interferon regulatory factor-1 is required for interferon-gamma-induced MHC class I genes in astrocytes. Journal of neuroimmunology. 2002;122(1–2):74–84. Epub 2002/01/05. PubMed . 11777545 10.1016/s0165-5728(01)00467-2

[43] Barbash ZS, Weissman JD, Campbell JA Jr., Mu J, Singer DS. Major histocompatibility complex class I core promoter elements are not essential for transcription in vivo. Molecular and cellular biology. 2013;33(22):4395–407. doi: 10.1128/MCB.00553-13 ; PubMed Central PMCID: PMC3838175. 24019072 10.1128/MCB.00553-13 PMC3838175

[44] Schieber M, Chandel NS. ROS function in redox signaling and oxidative stress. Current biology: CB. 2014;24(10):R453–62. Epub 2014/05/23. doi: 10.1016/j.cub.2014.03.034 ; PubMed Central PMCID: PMCPmc4055301. 24845678 10.1016/j.cub.2014.03.034 PMC4055301

[45] D'Autreaux B, Toledano MB. ROS as signalling molecules: mechanisms that generate specificity in ROS homeostasis. Nature reviews Molecular cell biology. 2007;8(10):813–24. Epub 2007/09/13. doi: 10.1038/nrm2256 . 17848967 10.1038/nrm2256

[46] Perier A, Fregni G, Wittnebel S, Gad S, Allard M, Gervois N, et al Mutations of the von Hippel-Lindau gene confer increased susceptibility to natural killer cells of clear-cell renal cell carcinoma. Oncogene. 2011;30(23):2622–32. Epub 2011/01/25. doi: 10.1038/onc.2010.638 . 21258414 10.1038/onc.2010.638

[47] Iliopoulos O, Kibel A, Gray S, Kaelin WG Jr. Tumour suppression by the human von Hippel-Lindau gene product. Nature medicine. 1995;1(8):822–6. Epub 1995/08/01. PubMed . 7585187 10.1038/nm0895-822

