# Supplementary figures and images for: Hypoxia and hypoxia-inducible factor (HIF) downregulate antigen-presenting MHC class I molecules limiting tumor cell recognition by T cells
Source: PLoS One. 2017 Nov 20;12(11):e0187314. doi: 10.1371/journal.pone.0187314 (PMC5695785; doi:10.1371/journal.pone.0187314)

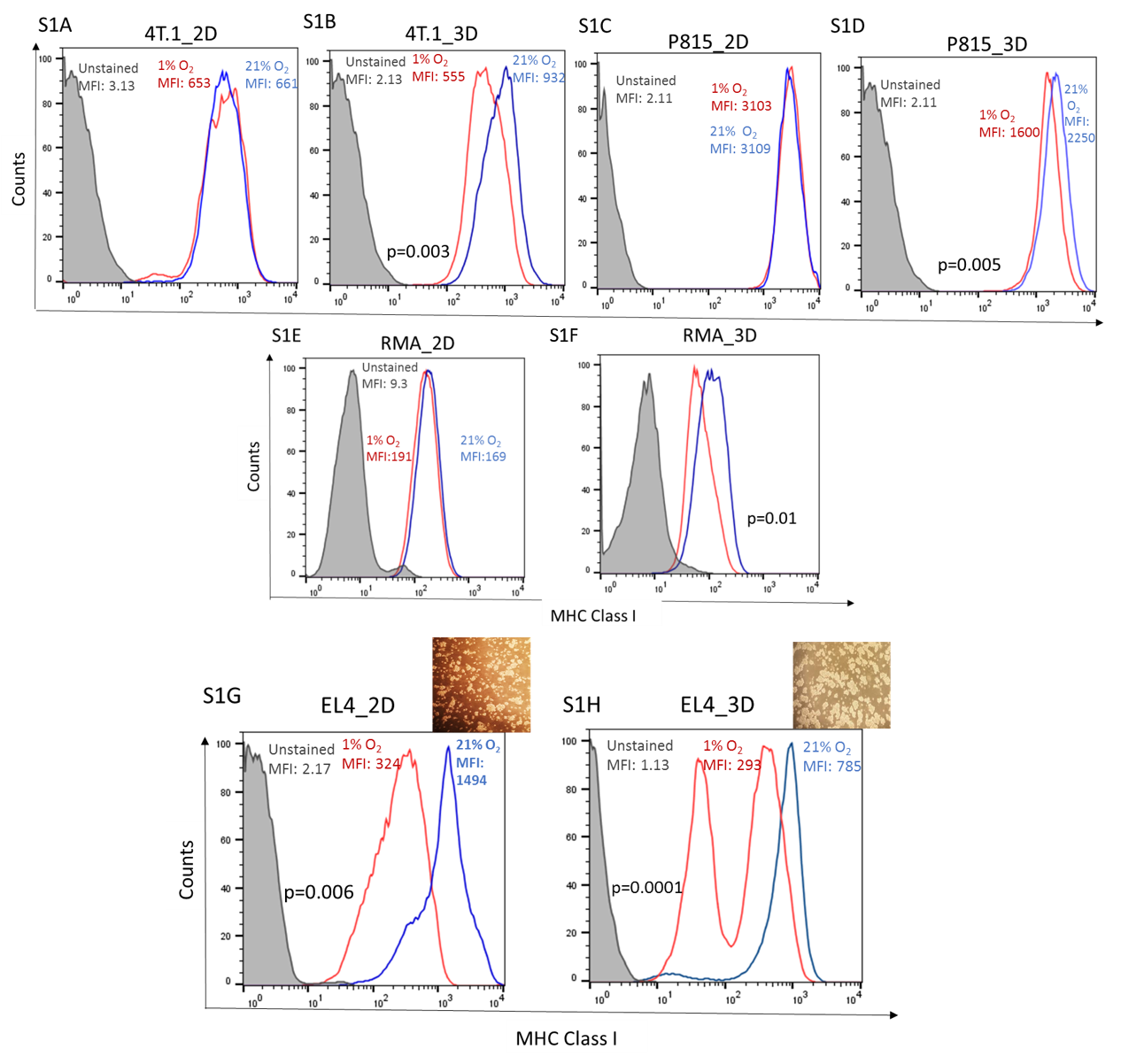

Supplement: S1 Fig — 4T.1 breast carcinoma (S1 A,B), P815 mastocytoma (S1 C,D), RMA T lymphoma (S1 E,F) and EL4 thymoma (S1 G,H) were cultured as 2D monolayers (indicated as 2D) or as 3D spheroids (indicated as 3D) and cultured under 21% O2 or 1% O2 for 48h. Levels of MHC class I expression was determined using flow cytometry. Representative histograms of 4 independent experiments are shown. Grey filled: unstained control; red: hypoxia; blue: normoxia. MFI: mean fluorescence intensity. Inset of S1 G,H: 40X magnification of El4 cells grown at 1% O2 in 2D culture (G) or in 3D culture (H) representing the growth of these cells in clumps irrespective of the culture format. (TIF) [file pone.0187314.s001.tif]

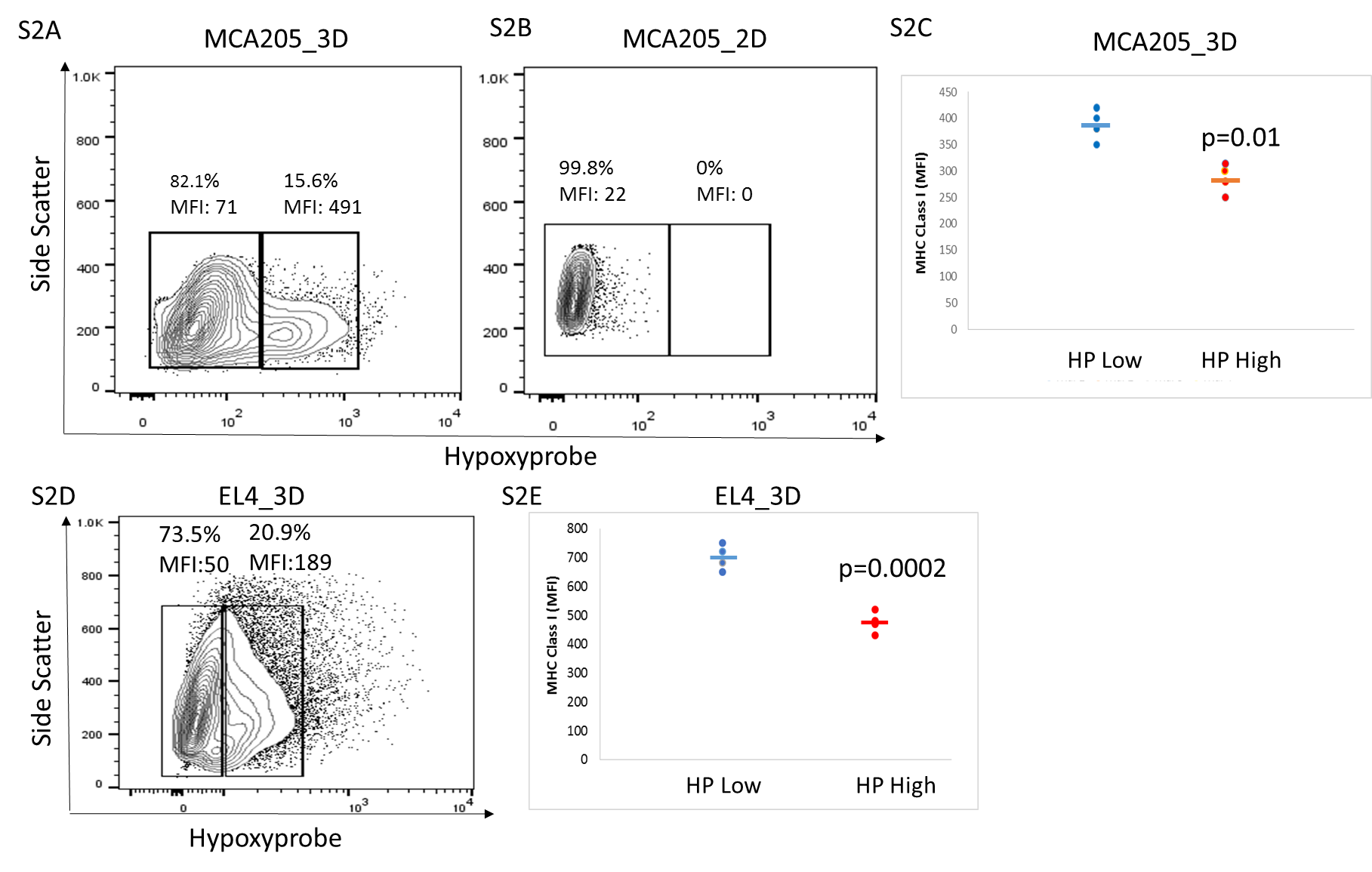

Supplement: S2 Fig — MCA-205 fibrosarcoma cells were grown as spheroids (S2A) or flat monolayer (S2B) in normoxic conditions (21% oxygen) and levels of hypoxia in each culture system assessed using hypoxyprobe. About 16% of the population was hypoxic in the 3D spheroids whereas there was no detectable levels of hypoxia in the 2D cultured cells. In 3D spheroids of EL4, about 20% of the population was hypoxic (S2D). Representative contour plots of three independent experiments shown. (S2C, E) Mean fluorescent intensity (MFI) of MHC class I expression on 3D spheroids (MCA205; S2C, EL4; S2E) from less hypoxic (HP low) and more hypoxic (HP high) regions showed inverse correlation between hypoxia and MHC class I expression. Each point on graph represents an independent experiment with the average represented as a dash. (TIF) [file pone.0187314.s002.tif]

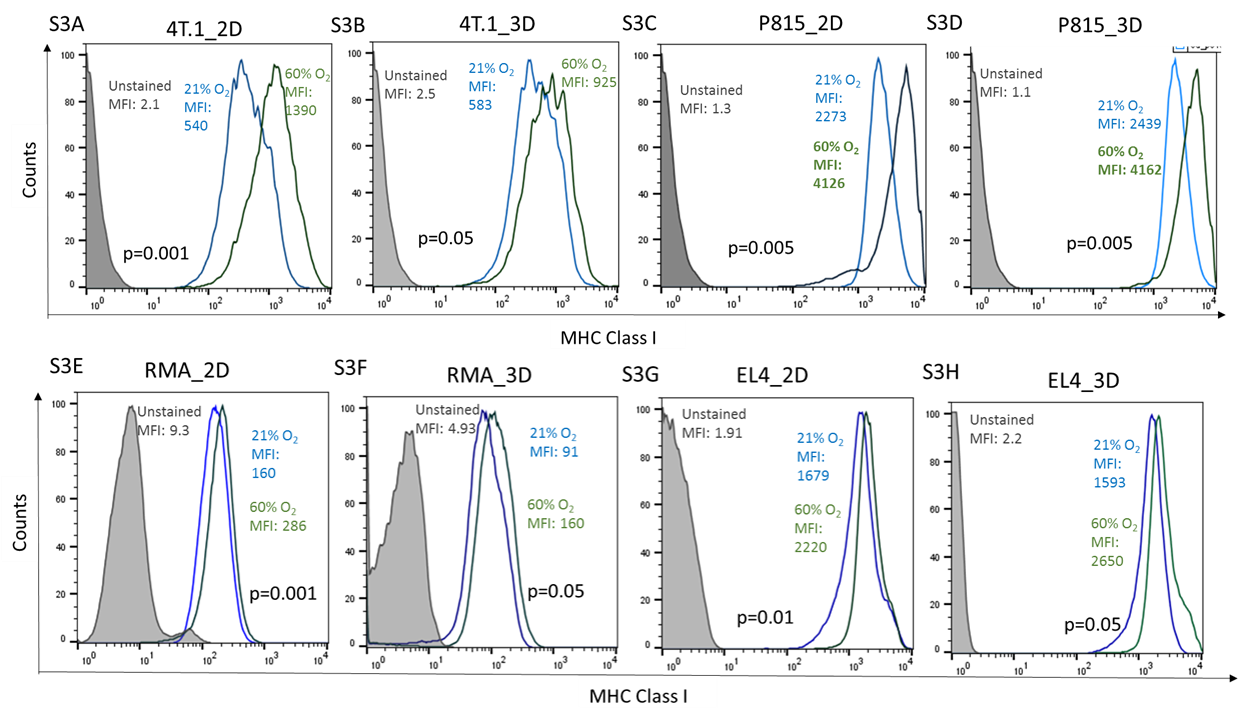

Supplement: S3 Fig — 4T.1 breast carcinoma (S3 A,B), P815 mastocytoma (S3 C,D), RMA T lymphoma (S3 E,F) and EL4 thymoma (S3 G,H) were cultured as 2D monolayers (indicated as 2D) or as 3D spheroids (indicated as 3D) and cultured under 21% O2 or 60% O2 for 48h. Levels of MHC class I expression was determined using flow cytometry. Representative histograms of 4 independent experiments are shown. Grey filled: unstained control; blue: normoxia; green: hyperoxia. MFI: mean fluorescence intensity. (TIF) [file pone.0187314.s003.tif]

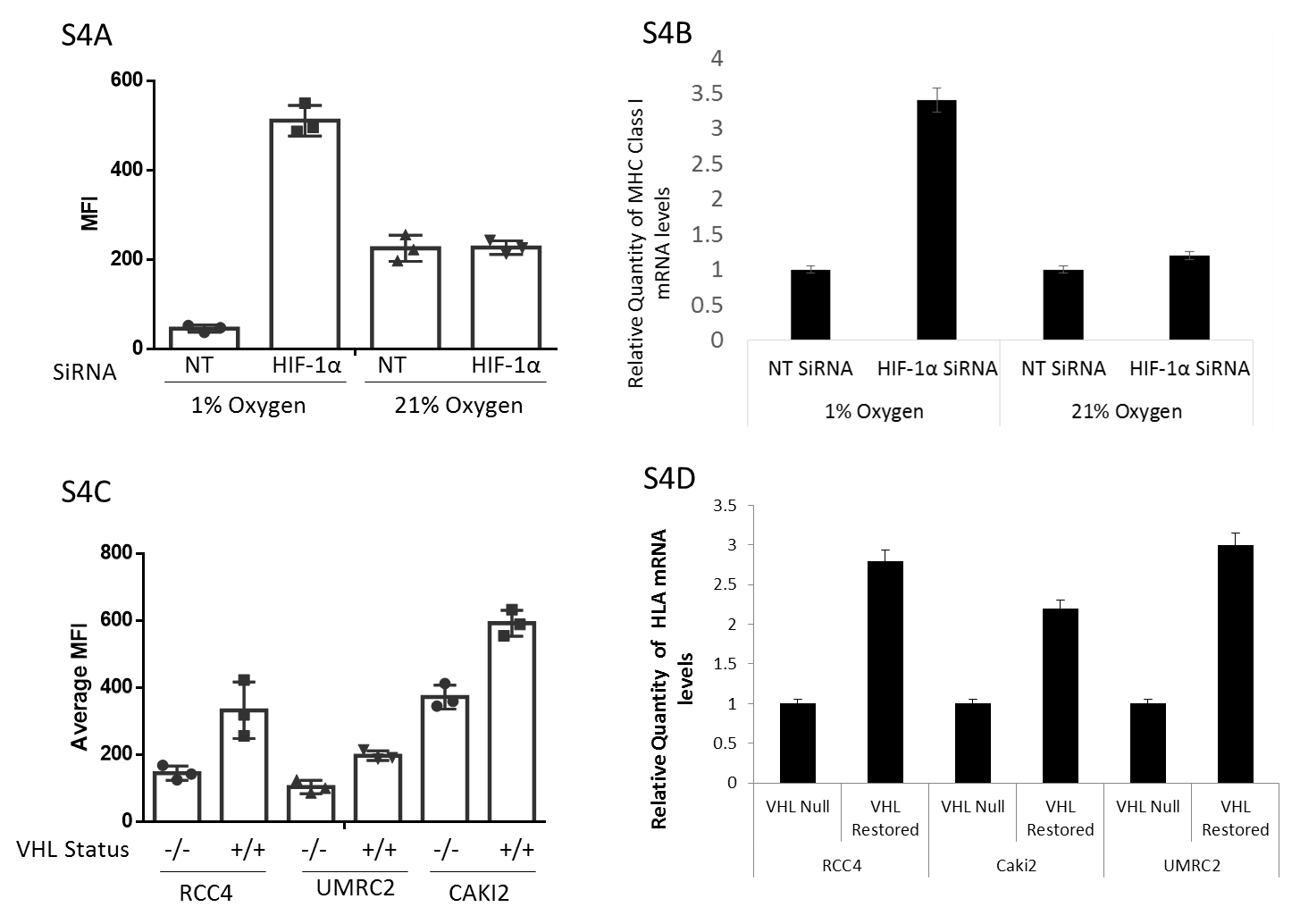

Supplement: S4 Fig — (SA, B): siRNA mediated knockdown of HIF-1α reversed hypoxic downregulation of MHC class I expression as compared with the scrambled, non-targeting (NT) siRNA control. MCA205 tumor cells were reverse transfected with scrambled siRNA (NT) or with HIF-1α specific siRNA and cultured as 3D spheroids under 1% or 21% oxygen for 48h. Levels of MHC class I surface expression was determined using flow cytometry; quantitative analysis of representative histograms shown in Fig 6 shown here (A). Levels of MHC class I transcripts were assessed using RT-qPCR (B). Average data of 3 independent experiments are shown. (S4C, D): Flow cytometry assessment of surface expression of HLA-ABC (S4C) and RT-qPCR analysis of HLA-ABC transcript levels (S4D) on paired isogenic renal cell carcinoma cell lines RCC4, UMRC2 and CAKI2. Each pair had the parental cell line that lacked endogenous wild-type VHL (VHL null, transfected with empty vector) and one with vector stably expressing functional VHL (VHL restored). Restoring VHL function and thereby reducing HIF expression, significantly increased HLA-ABC surface expression and transcript levels in the cells. Average data of 4 independent experiments are shown. (TIF) [file pone.0187314.s004.tif]

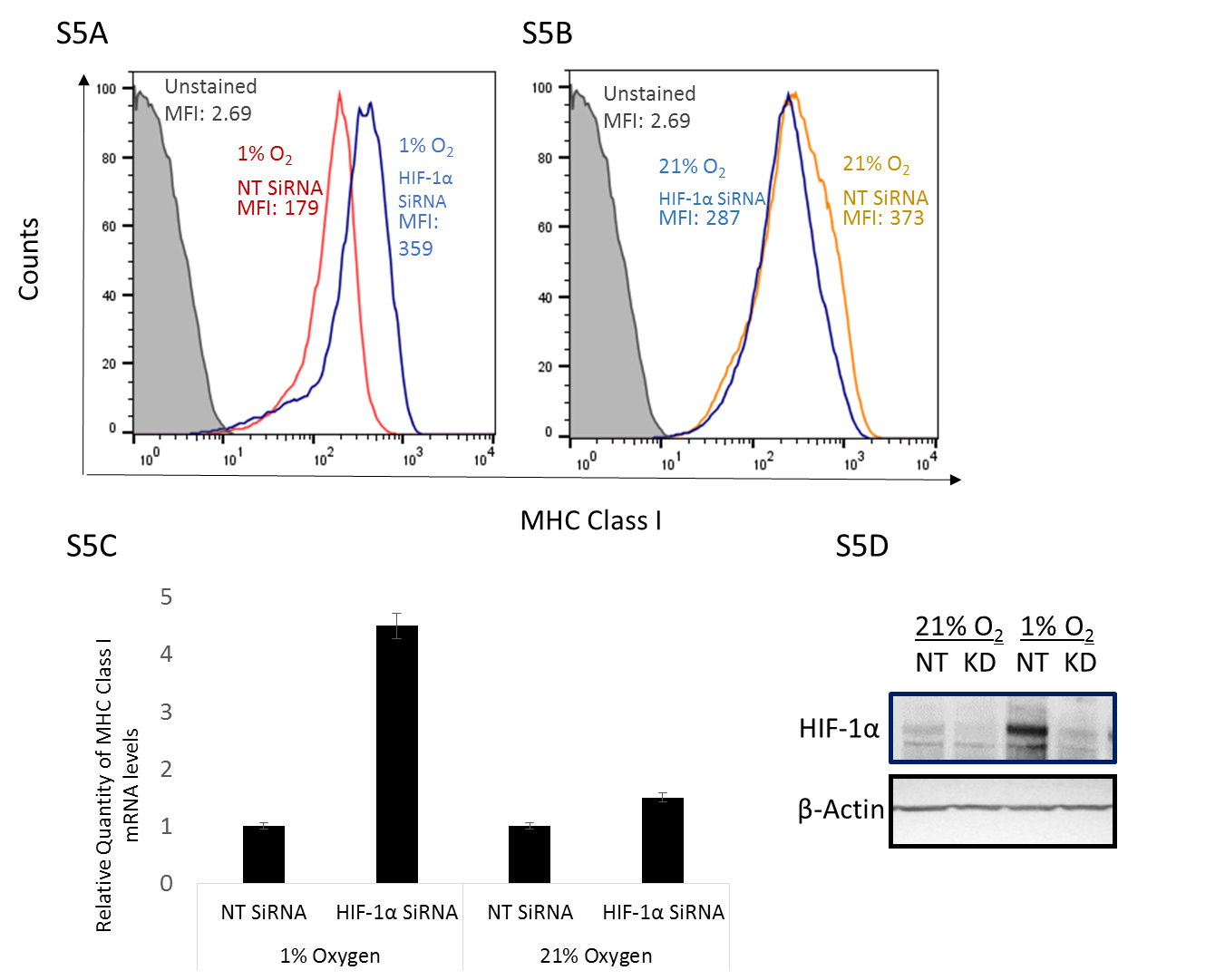

Supplement: S5 Fig — siRNA mediated knockdown of HIF-1α reversed hypoxic downregulation of MHC class I expression as compared with the scrambled, non-targeting (NT) siRNA control. EL4 tumor cells were reverse transfected with scrambled siRNA (NT; Red histogram) or with HIF-1α specific siRNA (blue histogram) and cultured as 3D spheroids under 1% (S5A) or 21% (S5B) oxygen for 48h. Levels of MHC class I surface expression was determined using flow cytometry (S5A,B). RT-qPCR was used to analyze MHC class I transcript levels. Ribosomal protein L32 was used as internal control (S5C). Efficacy of gene knockdown was assessed using western blot (S5D). β-actin was used as the loading control. Representative data of 2 independent experiments are shown. (PNG) [file pone.0187314.s005.png]

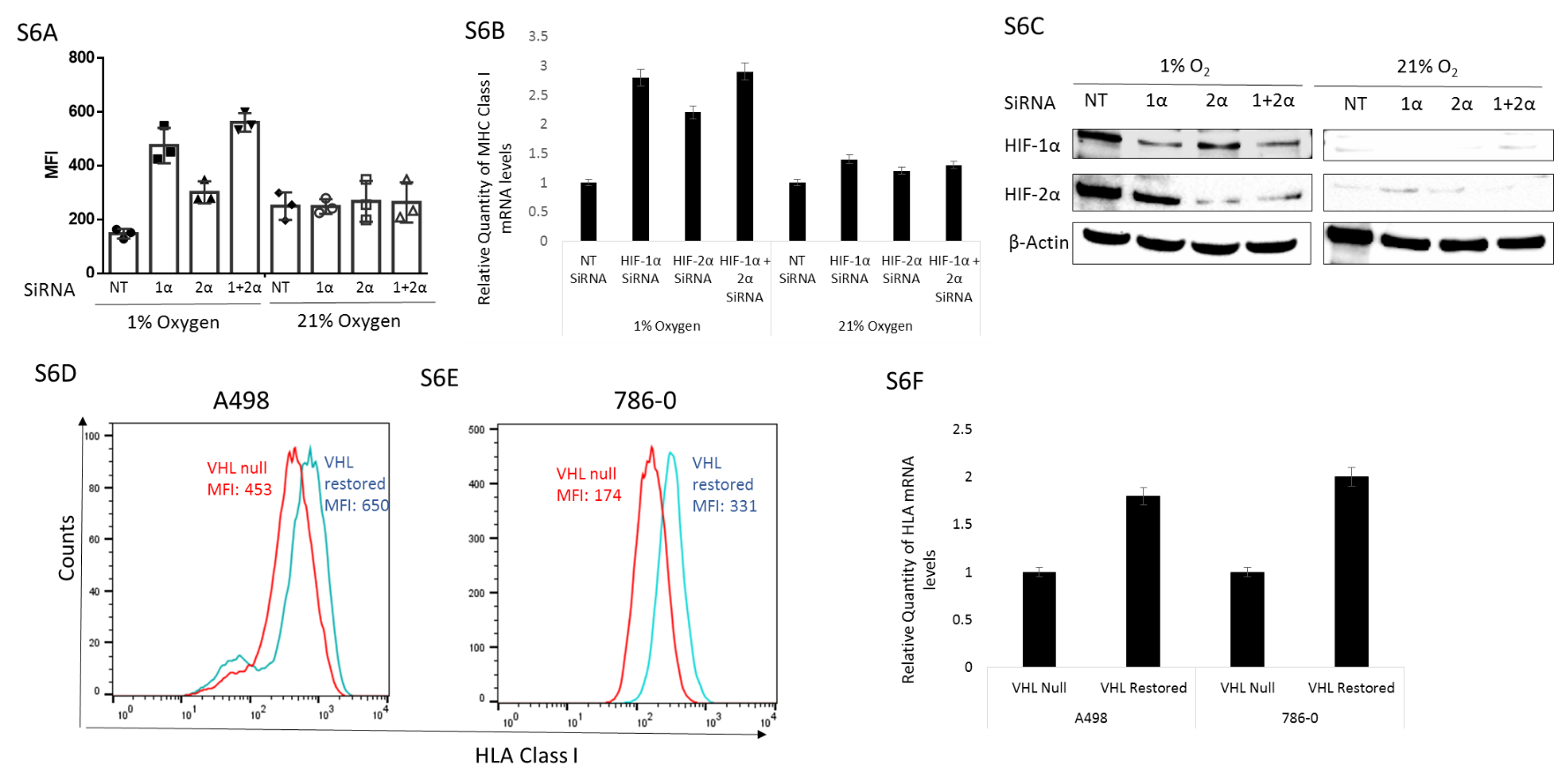

Supplement: S6 Fig — (S6A-C): siRNA mediated knockdown of HIF-1α, HIF-2α or both reversed hypoxic downregulation of MHC class I expression as compared with the scrambled, non-targeting (NT) siRNA control. MCA205 tumor cells were reverse transfected with scrambled siRNA (NT) or with HIF-1α, HIF-2α or both HIF-1α and HIF-2α specific siRNA and cultured as 3D spheroids under 1% or 21% oxygen for 48h. Levels of MHC class I surface expression was determined using flow cytometry (S6A). Transcripts levels were determined by RT-qPCR, with Ribosomal protein L32 as internal control (S6B). Efficacy of gene knockdown was assessed using western blot (S6C). Greater than 90% knockdown of HIF-1α/ HIF-2α was achieved. β-actin was used as the loading control. Average data of 3 independent experiments are shown. (S6D-F): Flow cytometry assessment of surface expression of HLA-ABC on paired isogenic renal cell carcinoma cell lines A-498 (D) and 786–0 (E). Each pair had the parental cell line that lacked endogenous wild-type VHL (VHL null, transfected with empty vector) and one with vector stably expressing functional VHL (VHL restored). Restoring VHL function and thereby reducing HIF expression, significantly increased HLA-ABC expression on the cells (S6D,E) and transcript levels (S6F). Representative histograms of 4 independent experiments are shown. Grey filled: Unstained control; Red: VHL null phenotype; Blue: VHL restored phenotype. For RT-qPCR, average data from 4 independent experiments shown. (TIF) [file pone.0187314.s006.tif]

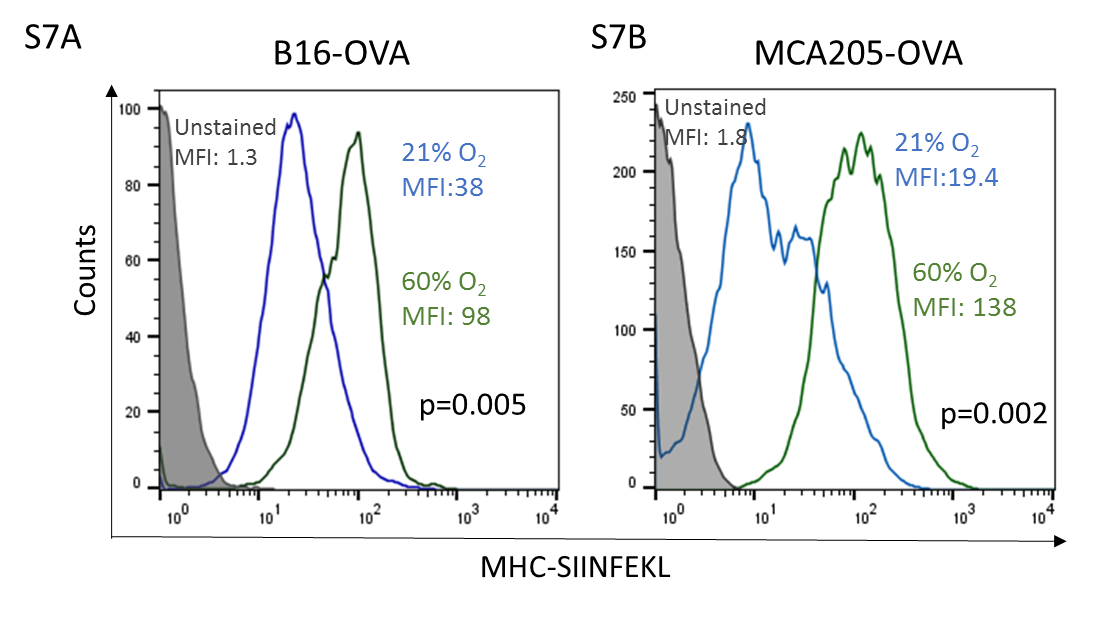

Supplement: S7 Fig — (S7A,B) 60% O2 significantly upregulated expression of the immunodominant peptide of ovalbumin, SIINFEKL. B16 melanoma cells (S7A) or MCA205 fibrosarcoma cells (S7B) transfected with ovalbumin were cultured as 3D spheroids for 48h either under 21% or 60% O2. Levels of MHC class I-SIINFEKL surface expression was determined using flow cytometry, with overnight incubation at 4°C with the mAb to MHC-SIINFEKL. Representative histograms of 4 independent experiments are shown. Grey filled: unstained control; blue: normoxia; green: hyperoxia. MFI: mean fluorescent intensity. (TIF) [file pone.0187314.s007.tif]
